# Glutamate Secretion and Metabotropic Glutamate Receptor 1 Expression during Kaposi's Sarcoma-Associated Herpesvirus Infection Promotes Cell Proliferation

**DOI:** 10.1371/journal.ppat.1004389

**Published:** 2014-10-09

**Authors:** Mohanan Valiya Veettil, Dipanjan Dutta, Virginie Bottero, Chirosree Bandyopadhyay, Olsi Gjyshi, Neelam Sharma-Walia, Sujoy Dutta, Bala Chandran

**Affiliations:** H. M. Bligh Cancer Research Laboratories, Department of Microbiology and Immunology, Chicago Medical School, Rosalind Franklin University of Medicine and Science, North Chicago, Illinois, United States of America; University of Pennsylvania Medical School, United States of America

## Abstract

Kaposi's sarcoma associated herpesvirus (KSHV) is etiologically associated with endothelial Kaposi's sarcoma (KS) and B-cell proliferative primary effusion lymphoma (PEL), common malignancies seen in immunocompromised HIV-1 infected patients. The progression of these cancers occurs by the proliferation of cells latently infected with KSHV, which is highly dependent on autocrine and paracrine factors secreted from the infected cells. Glutamate and glutamate receptors have emerged as key regulators of intracellular signaling pathways and cell proliferation. However, whether they play any role in the pathological changes associated with virus induced oncogenesis is not known. Here, we report the first systematic study of the role of glutamate and its metabotropic glutamate receptor 1 (mGluR1) in KSHV infected cell proliferation. Our studies show increased glutamate secretion and glutaminase expression during *de novo* KSHV infection of endothelial cells as well as in KSHV latently infected endothelial and B-cells. Increased mGluR1 expression was detected in KSHV infected KS and PEL tissue sections. Increased c-Myc and glutaminase expression in the infected cells was mediated by KSHV latency associated nuclear antigen 1 (LANA-1). In addition, mGluR1 expression regulating host RE-1 silencing transcription factor/neuron restrictive silencer factor (REST/NRSF) was retained in the cytoplasm of infected cells. KSHV latent protein Kaposin A was also involved in the over expression of mGluR1 by interacting with REST in the cytoplasm of infected cells and by regulating the phosphorylation of REST and interaction with β-TRCP for ubiquitination. Colocalization of Kaposin A with REST was also observed in KS and PEL tissue samples. KSHV infected cell proliferation was significantly inhibited by glutamate release inhibitor and mGluR1 antagonists. These studies demonstrated that elevated glutamate secretion and mGluR1 expression play a role in KSHV induced cell proliferation and suggest that targeting glutamate and mGluR1 is an attractive therapeutic strategy to effectively control the KSHV associated malignancies.

## Author Summary

Kaposi's sarcoma associated herpesvirus (KSHV), prevalent in immunosuppressed HIV infected individuals and transplant recipients, is etiologically associated with cancers such as endothelial Kaposi's sarcoma (KS) and B-cell primary effusion lymphoma (PEL). Both KS and PEL develop from the unlimited proliferation of KSHV infected cells. Increased secretion of various host cytokines and growth factors, and the activation of their corresponding receptors, are shown to be contributing to the proliferation of KSHV latently infected cells. Glutamate, a neurotransmitter, is also involved in several cellular events including cell proliferation. In the present study, we report that KSHV-infected latent cells induce the secretion of glutamate and activation of metabotropic glutamate receptor 1 (mGluR1), and KSHV latency associated LANA-1 and Kaposin A proteins are involved in glutaminase and mGluR1 expression. Our functional analysis showed that elevated secretion of glutamate and mGluR1 activation is linked to increased proliferation of KSHV infected cells and glutamate release inhibitor and glutamate receptor antagonists blocked the proliferation of KSHV infected cells. These studies show that proliferation of cancer cells latently infected with KSHV in part depends upon glutamate and glutamate receptor and therefore could potentially be used as therapeutic targets for the control and elimination of KSHV associated cancers.

## Introduction

Kaposi's sarcoma-associated herpesvirus or human herpesvirus-8 (KSHV/HHV-8) infection is etiologically associated with Kaposi's sarcoma (KS), a vascular endothelial tumor, and two B-cell lymphoproliferative diseases, primary effusion lymphoma (PEL) or body-cavity based lymphoma (BCBL) and multicentric Castleman's disease [1], [2], [3]. These cancers occur more frequently in the setting of immunosuppression, including HIV-1 infected patients, and develop from cells latently infected with KSHV. *In vivo* KSHV has a broad tropism and viral genome and transcripts are detected in a variety of cells such as B cells, endothelial cells, monocytes, keratinocytes, and epithelial cells [4], [5]. Latent KSHV DNA is present in vascular endothelial and spindle cells of KS lesions, associated with expression of latency associated ORF73 (LANA-1), ORF72 (v-cyclin D), K13 (v-FLIP), and K12 (Kaposin) genes and microRNAs [5]. Cell lines with B cell characteristics, such as BC-1, BC-3, BCBL-1, HBL-6 and JSC have been established from PEL tumors [4], [5]. In PEL cells, in addition to the above set of latent genes, the K10.5 (LANA-2) gene is also expressed [4], [5]. About 1–3% of PEL cells spontaneously enter the lytic cycle and virus induced from these cells by chemicals serve as the source of virus. Multiple genome copies of both KSHV and EBV exist in latent form in BC-1, HBL-6 and JSC cells while BCBL-1 and BC-3 cells carry only the KSHV genome [4], [5]. KSHV infects a wide variety of human cell types *in vitro*, including fibroblasts, keratinocytes, B cells, endothelial, and epithelial cells [4], [5], [6]. Following infection, KSHV establishes latency within the target cells and the expression of the viral latent ORF71, ORF72, ORF73, and K13 genes continues to maintain latency [7]. In addition, host genes required for regulating apoptosis, signal induction, cell cycle regulation, inflammatory response, and angiogenesis are also highly upregulated in the latently infected cells [8].

Studies have linked the expression of KSHV latency genes ORF71 (v-FLIP), -72 (v-Cyclin), -73 (LANA-1) and K12 (Kaposin A) to the oncogenic activity of latently infected cells [9], [10], [11]. These genes induce the oncogenic potential of KSHV by increasing proliferative potential, growth and chromosome instability as well as by preventing apoptosis of the infected cells [9], [10], [11]. A hallmark of KSHV associated cancers is the excessive secretion of cytokines and growth factors [12], [13], [14]. Modulation by viral proteins and virally induced cellular proteins promote the secretion of autocrine and paracrine cytokines and growth factors leading into the proliferation, survival, and growth of the latently infected cells [12], [13], [15], [16], [17], [18]. However, the mechanism behind KSHV induced cancer progression is not completely understood.

Glutamate is a major excitatory neurotransmitter in the mammalian brain. It also plays a central role in several cellular functions, including cell survival and death by its interaction with receptors [19]. Glutamate released into the extracellular space binds and activates two classes of cell surface receptors, ionotropic (iGluRs) and G protein-coupled metabotropic glutamate receptors (mGluRs). There are three groups of mGluRs, and group I mGluRs have been extensively studied in relation to cell survival and death. Group I consists of mGluR1 and mGluR5 subtypes (mGluR1/5) which are coupled to Gα_q/11_ proteins. Agonist stimulation of group I mGluRs activate PLC, which results in the activation of PKC, PKC dependent pathways, and ERK1/2 [20], [21], [22]. The group I mGluRs are distributed in a variety of non-neuronal cells including human B and microvascular dermal endothelial cells, the natural target cells of KSHV [23], [24], [25]. However, the involvement of excess glutamate secretion and glutamate receptor expression in cell proliferation is an unexplored area of research in the KSHV oncogenesis field. This current study was undertaken with a rationale that identifying and defining the role of glutamate in KSHV biology will lead into targeted specific treatments for KSHV-associated malignancies.

Our studies demonstrate that KSHV infection induces the secretion of glutamate and expression of mGluR1 receptor, and increased mGluR1 expression was detected in KS and PEL tissue sections. Most notably, glutamate secretion and mGluR1 activation in KSHV latently infected cells occurred through two independent pathways regulated by two individual viral latent proteins, LANA-1 and Kaposin A. Our data highlight how KSHV LANA-1 and Kaposin A proteins contribute to the generation of glutamate, activation of mGluR1, and strongly suggest the possibility of exploiting the glutamatergic system for the therapeutic intervention of KSHV dependent cancers.

## Results

### KSHV infection induces glutamate secretion

**Figure 1:**
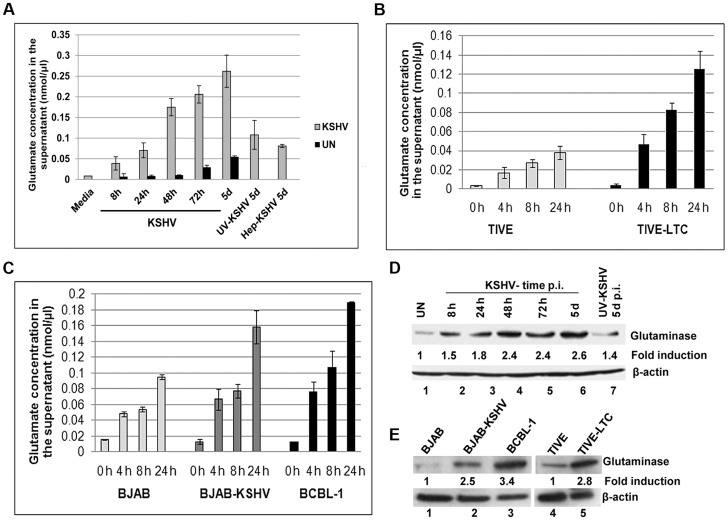
Detection of increased glutamate secretion and glutaminase expression during *de novo* KSHV infection and in latently infected cells. A) Primary HMVEC-d cells were left uninfected (UN) or infected with KSHV for different time points or with 100 µg heaprin-treated KSHV (Hep-KSHV) or UV-KSHV for 5d. Supernatants from uninfected and infected cells were collected and measured for the release of glutamate by a glutamate assay kit. B) Glutamate release in supernatants collected at various times from TIVE, TIVE-LTC, (C) BJAB, BJAB-KSHV, and BCBL-1 cells. Concentration of glutamate released is expressed in nanomoles per microliter. In a–c, error bars represent the mean ± SD of three independent experiments. D) Expression of glutaminase assessed by immunoblot analysis in primary HMVEC-d cells left uninfected or infected with KSHV for the indicated time points or UV-KSHV for 5 d. (E) Glutaminase expression in TIVE, TIVE-LTC, BJAB, BJAB-KSHV and BCBL-1 cells. β-actin was used as loading control. Fold change is relative to the uninfected control.

To determine the role of glutamate in KSHV infection, we first evaluated the secretion of glutamate during *de novo* KSHV infection of primary human microvascular dermal endothelial cells (HMVEC-d). Kinetics of glutamate secretion showed that KSHV infection robustly increased glutamate release as early as 8 h post-infection (p.i.) which continued to increase throughout the 5 d p.i. observation period (Figure 1A). In contrast, when the cells were infected with replication defective UV treated KSHV for 5d, there was no significant difference in glutamate secretion between uninfected and UV-KSHV infected cells, (Fig. 1A) suggesting that viral gene expression is required for the increased secretion of glutamate. To determine whether the secretion of glutamate is specifically induced by KSHV, cells were infected with KSHV pre-incubated with heparin (Hep-KSHV), which is known to block the binding and entry of KSHV to the target cells [26]. In contrast to the untreated virus, heparin treated virus (Hep-KSHV) considerably reduced the secretion of glutamate (Figure 1A). This suggested that KSHV entry and infection is required for the increased secretion of glutamate.

KS is an endothelial tumor, whereas PEL is of B-cell origin [2], [27], [28]. The telomerase immortalized endothelial cell line (TIVE) latently infected with KSHV (TIVE-LTC), and the PEL derived B-cell line BCBL-1 are well-established *in vitro* models to study KS and PEL, respectively [2], [29]. In addition, BJAB-KSHV, a Burkitt's lymphoma B-cell line carrying latent KSHV DNA, has also been used as an additional model for studying KSHV pathogenesis [30]. To test whether the process of glutamate generation is relevant to KS, we measured the secretion of glutamate in KSHV TIVE-LTC cells as well as in uninfected control TIVE cells. Similar to *de novo* KSHV infection, higher levels of glutamate release were observed in KSHV(+) TIVE-LTC cells than in KSHV(−) TIVE cells (Figure 1B). When the association of glutamate to PEL was assessed, high levels of glutamate release were observed in KSHV (+) BCBL-1 and BJAB-KSHV cells compared to the KSHV (−) B-cell line BJAB (Figure 1C).

### KSHV infection induces glutaminase expression

To elucidate the mechanisms of glutamate generation in the infected cells, we next determined the expression of glutaminase, the major enzyme responsible for glutamate production [31]. Compared to the uninfected cells, a time dependent increase in glutaminase expression was observed during 8 h, 24 h, 48 h and 5 d of *de novo* infection of primary HMVEC-d cells by KSHV (Figure 1D, lanes 1–6). In contrast, at 5 d p.i. with UV-KSHV, no significant difference in glutaminase expression from uninfected cells was observed (Figure 1D, lanes 1 and 7). These results demonstrated that the increased glutamate secretion is linked with increased glutaminase expression in the infected cells. This link was further confirmed by the detection of a higher level of glutaminase expression in the latently infected TIVE-LTC (2.8 fold), BJAB-KSHV (2.5 fold), and BCBL-1 cells (3.4 fold) than in their respective uninfected control TIVE and BJAB cells (Figure 1E, lanes 1–5).

To further investigate the role of glutaminase in glutamate secretion, we used a glutaminase specific inhibitor, L-DON (6-diazo-5-oxo-norleucine) [32]. Cells were treated with L-DON at a concentration of 500 µM and 1 mM and the supernatants were analyzed for glutamate release. We found that 500 µM of L-DON inhibited glutamate secretion by >50% and 1 mM of L-DON by >65%. Dose dependent inhibition of glutamate secretion in L-DON treated cells strongly suggested that glutaminase is the major enzyme that contributes to the generation of excess glutamate in KSHV infected cells (Figure S1A). L-DON had no significant cytotoxicity on BJAB cells at 500 µM and 1 mM concentrations (data not shown).

### KSHV LANA-1 mediated c-Myc activation plays a role in glutamate secretion and glutaminase expression

**Figure 2:**
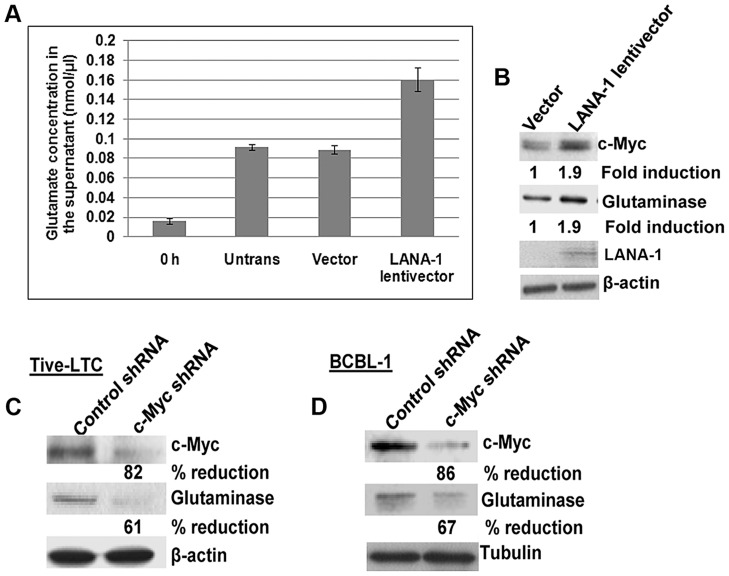
Exogenous KSHV latency associated LANA-1 protein expression increases glutamate secretion and the expression of glutaminase and c-Myc in BJAB cells. (**A**) BJAB cells were transduced with empty lentivirus vector or lentivirus LANA-1 constructs. 72 h after transduction, the media was replaced with fresh medium and cultured for a further 24 h; supernatants were collected and measured for the release of glutamate. Error bars represent the mean ± SD of three independent experiments. (**B**) Cell lysates from the transduced cells were Western blotted for glutaminase, LANA-1, and c-Myc expression. β-actin was used as loading control. Fold change is relative to the vector control. (**C, D**) Western blot analysis of c-Myc and glutaminase expression in TIVE-LTC (**C**) and BCBL-1 cells (**D**) following transduction with c-Myc shRNA or control shRNA.

KSHV latency-associated ORF73 gene product LANA-1 has been shown to induce c-Myc expression [33]. Since c-Myc has also been shown to activate the expression of glutaminase [34], we hypothesized that the increased glutamate secretion observed in KSHV infected cells could be mediated by LANA-1 through its c-Myc activation, which in turn stimulates the expression of glutaminase. To test this hypothesis, when BJAB cells were transduced with lentivirus constructs of LANA-1, we observed increased secretion of glutamate in LANA-1 transduced cells compared to vector alone (Figure 2A). We also observed ∼2-fold increase in c-Myc and glutaminase protein expression in LANA-1 transduced cells (Figure 2B).

To support our finding that LANA-1 mediated c-Myc activation is directly involved in glutaminase expression and glutamate secretion, we used lentiviruses encoding shRNAs to knock down c-Myc in BJAB cells over expressing LANA-1. As shown in figure S1B, LANA-1 over expression induced the secretion of glutamate, and this induction was abolished by the knockdown of c-Myc (Figure S1B). Since no considerable increase in glutamate release was observed in the absence of c-Myc in LANA-1 expressing cells (Figure S1B), these results suggested that LANA-1 mediated c-Myc activation is required for glutamate release.

To confirm the functional relationship of c-Myc expression with glutaminase expression in KSHV infected cells, we transduced TIVE-LTC cells and BCBL-1 cells with c-Myc and control shRNA lentiviral vectors. A significant reduction in glutaminase expression was observed in c-Myc knockdown TIVE-LTC cells (61%) and BCBL-1 cells (67%) compared to control shRNA transduced cells (Figures 2C and D). These results suggested that LANA-1 mediated c-Myc activation plays a crucial role in the expression of glutaminase and glutamate secretion in cells latently infected with KSHV.

### mGluR1 expression is upregulated in KSHV infected cells

**Figure 3:**
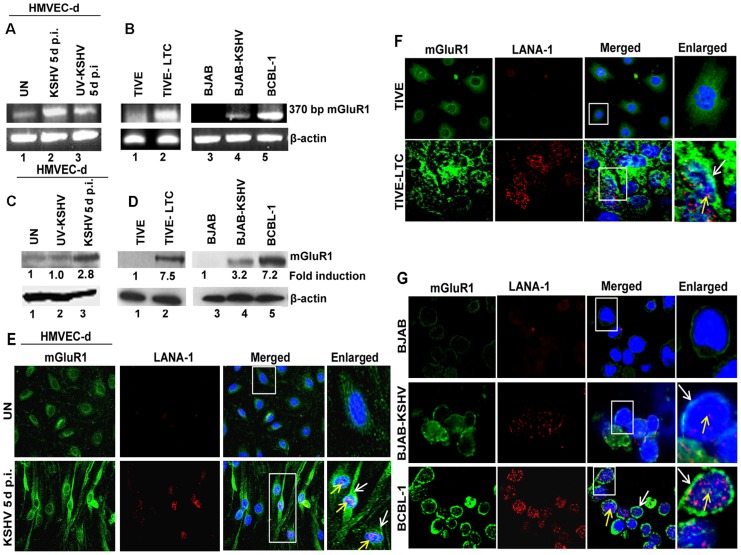
mGluR1 expression is upregulated in KSHV infected cells. (A) RNA isolated from uninfected HMVEC-d or infected with either live KSHV or UV-KSHV for 5 d were analyzed for mGluR1 gene expression by RT-PCR. β-actin was used as an internal control. B) RT-PCR for mGluR1 expression in RNA isolated from TIVE, TIVE-LTC, BJAB, BJAB-KSHV and BCBL-1 cells. C) Western blot determination of mGluR1 expression in HMVEC-d cells left uninfected or infected with KSHV and UV-KSHV for 5 d. (D) Western blot for mGluR1 expression in TIVE and TIVE-LTC cells, BJAB, BJAB-KSHV and BCBL-1 cells. Fold induction was calculated relative to the uninfected control. (E, F, G) mGluR1 expression in KSHV infected cells determined by double immunostaining with mGluR1 and LANA-1 in uninfected and KSHV infected HMVEC-d cells (Magnifications 40×) (E), TIVE and TIVE-LTC cells (Magnifications 40×) (F), and BJAB, BJAB-KSHV and BCBL-1 cells (G) (Magnifications 80×). Boxed areas are enlarged. White arrows indicate mGluR1 staining and yellow arrows indicate LANA-1 staining. Nuclei were stained with DAPI.

Among the several types of glutamate receptors, mGluR1 is considered an oncogenic protein due to its ability to regulate the functions related to cancer cell proliferation [35], [36]. Hence, we theorized that the biological effect of glutamate in latent KSHV induced oncogenesis may be mediated through the expression of mGluR1 receptors. To test this, we first determined mGluR1 expression by RT-PCR in primary endothelial cells infected for 5 d with KSHV and UV-KSHV. Compared to uninfected cells, KSHV infection increased the expression of mGluR1 (Figure 3A). In contrast, UV treated virus had no significant effect on mGluR1 expression (Figure 3A) and suggested that sustained mGluR1 receptor expression probably depended upon KSHV gene expression. When the relative expression levels for the mGluR1 receptor in KSHV latent TIVE-LTC, BJAB-KSHV and BCBL-1 cells as well as control BJAB and TIVE cells were determined by RT-PCR, upregulation of mGluR1 in both KSHV (+) TIVE-LTC cells and BCBL-1 cells was observed compared to uninfected TIVE and BJAB cells (Figure 3B). Western blot (Figure 3C) and immunoprecipitation analysis (Figure S2A) confirmed the higher levels of mGluR1 protein in *de novo* KSHV infected primary cells compared to the uninfected and UV-KSHV infected cells. Similarly, high levels of mGluR1 expression were also observed in BJAB-KSHV, BCBL-1 and TIVE-LTC cell lines by Western blots (Figure 3D) and by immunoprecipitation analysis (Figure S2B).

The expression of mGluR1 in KSHV infected primary cells and latent cells was also examined by immunofluorescence assay (IFA). Increased mGluR1 staining was detected in LANA-1 expressing spindle shaped HMVEC-d cells infected with KSHV (Figure 3E), as well as in TIVE-LTC, BJAB-KSHV and BCBL-1 cells compared to their respective uninfected controls (Figures 3F and G). These results clearly demonstrated that KSHV infection results in the increased mGluR1 expression in latently infected cells.

### mGluR1 expression is up regulated in tissue samples of KS and PEL patients

**Figure 4:**
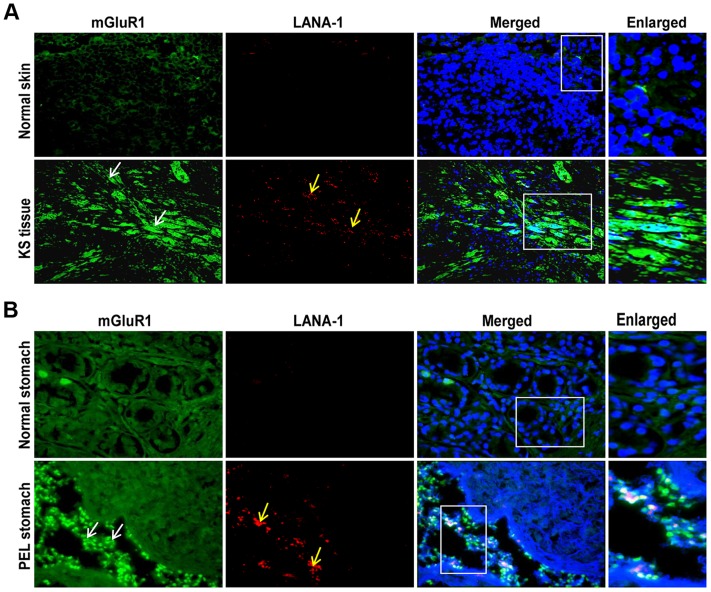
mGluR1 detection in KSHV infected patient samples. A) Immunofluorescence detection of mGluR1 and LANA-1 expression in normal human skin and KS tissue. B) Immunofluorescence staining of mGluR1 expression in normal stomach tissue and PEL affected stomach tissue. The tissue sections were counterstained with DAPI. White and yellow arrows indicate mGluR1 and LANA-1 staining, respectively. Magnifications 20×. Boxed areas are enlarged.

To verify the pathological association of mGluR1 in KSHV associated cancers, we immunostained normal as well as KSHV infected KS and PEL tissues by dual labeled IFA for mGluR1 and KSHV LANA-1 as a marker for infection. Strong positive immunostaining for both mGluR1 and LANA-1 were detected in the spindle shaped endothelial cells of KS tissue (Figure 4A), and in the stomach PEL (Figure 4B) samples. In contrast, only a basal level of mGluR1 was detected in control normal skin and stomach samples (Figures 4A and B). These results clearly demonstrated the *in vivo* association of increased mGluR1 expression with KSHV infection.

### mGluR1 expression regulating host RE-1 silencing transcription factor/neuron restrictive silencer factor (REST/NRSF) is retained in the cytoplasm of KSHV infected cells

The expression of mGluR1 in non-neuronal cells is regulated by RE-1 silencing transcription factor/neuron restrictive silencer factor (REST/NRSF) [37]. Binding of REST to a DNA recognition sequence called the neuron restrictive silencer elements (NRSE or RE-1) repress the expression of neuronal genes such as mGluR1 in non-neuronal cells [37], [38], [39]. To analyze whether REST expression plays any role in mGluR1 expression in KSHV infected cells, we determined the expression of REST mRNA and protein. real-time RT-PCR analysis of REST revealed similar levels of REST expression in both uninfected and KSHV infected latent cells (Figures S3A and B). However, REST protein expression determination by Western blots showed 55%, 72% and 42% reduction in BJAB-KSHV, BCBL-1 and TIVE-LTC cells, respectively, compared to the respective controls (Figures S3C and D). This suggested that REST expression in KSHV infected cells is probably modulated at the post-transcriptional level.

**Figure 5:**
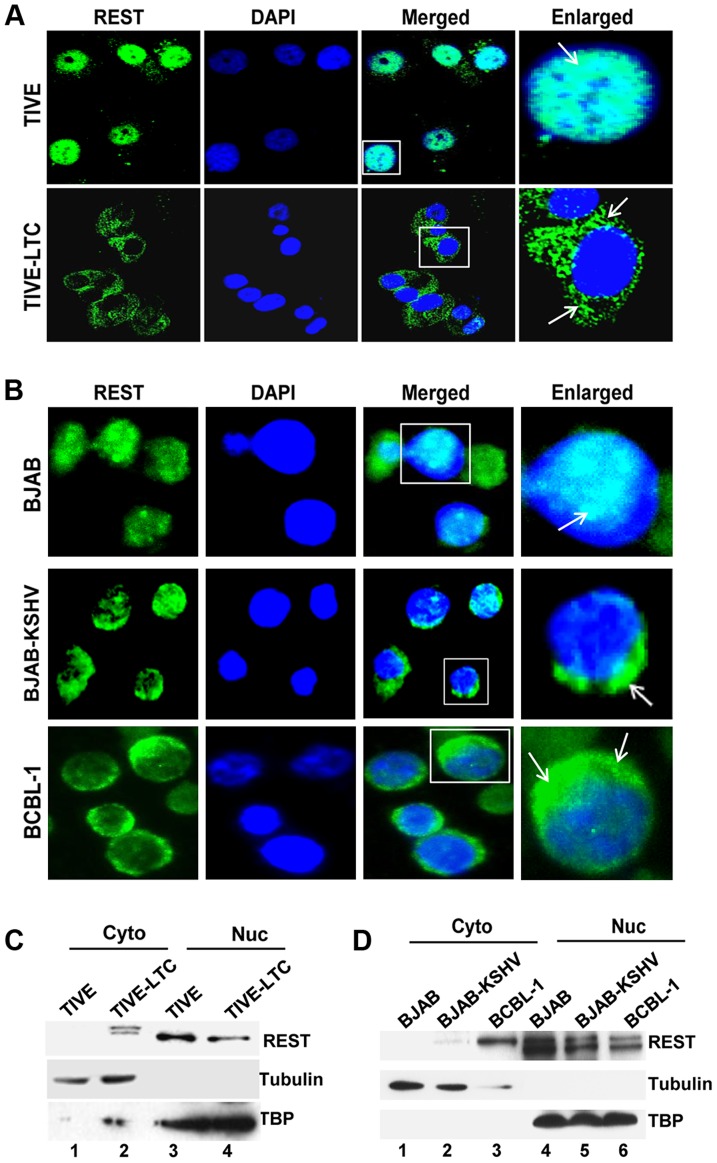
Cytoplasmic localization of REST in KSHV infected cells. A) Immunofluorescence staining showing nuclear and cytoplasmic localization of REST in TIVE and TIVE-LTC cells, (B) BJAB, BJAB-KSHV, and BCBL-1 cells. Nuclei were stained with DAPI. Boxed areas are enlarged. The arrows indicate REST localization. (C and D) Subcellular fractionation and REST detection. TIVE and TIVE-LTC (C), BJAB, BJAB-KSHV, and BCBL-1 cell (D) lysates were fractionated into nuclear and cytoplasmic fractions, and the fractions were Western blotted with REST antibodies. Nuclear and cytoplasmic fractions were blotted for TBP or tubulin to validate the purity of nuclear and cytoplasmic fractions, respectively.

To decipher the mechanism regulating REST expression at the post-translational level, we first determined the subcellular localization of REST in TIVE and TIVE-LTC cells by IFA. In the uninfected TIVE cells, REST was highly expressed and was predominantly localized in the nucleus (Figure 5A). In contrast, REST distribution was markedly decreased in the nucleus of TIVE-LTC cells and was predominantly localized to the cytoplasm (Figure 5A). A similar cytoplasmic relocalization of REST was observed in almost all KSHV infected BJAB-KSHV and BCBL-1 cells compared to the uninfected BJAB cells where it was exclusively localized in the nucleus (Figure 5B).

Western blot analysis of cytoplasmic and nuclear fractions of the KSHV positive cell lines confirmed that REST localization is significantly decreased in the nucleus (Figures 5C and D) with a concomitant increase in the cytoplasm of infected cells, whereas it was undetectable in the cytoplasm of uninfected TIVE and BJAB cells (Figures 5C and D, lane 1). Interestingly, analyses of REST in the cytoplasmic fractions from the infected cells showed a small shift in molecular weight in both TIVE-LTC (Figure 5C, lane 2) and BCBL-1 cells (Figure 5D, lane 3). We reasoned that this small shift in band size could be due to phosphorylation of REST, which is known to result in migration differences on SDS-PAGE. To determine whether the shifted band detected in the cytoplasm is indeed the phosphorylated form of REST, we first treated the cytoplasmic extracts from TIVE-LTC cells with lambda phosphatase or with lambda phosphatase and phosphatase inhibitor, and then the extracts were Western blotted. Treatment with lambda phosphatase resulted in the disappearance of the modified band, suggesting that the shift in band size was due to phosphorylation (Figure S4A).

### REST is phosphorylated and ubiquitinated via β-TRCP in KSHV infected cells

**Figure 6:**
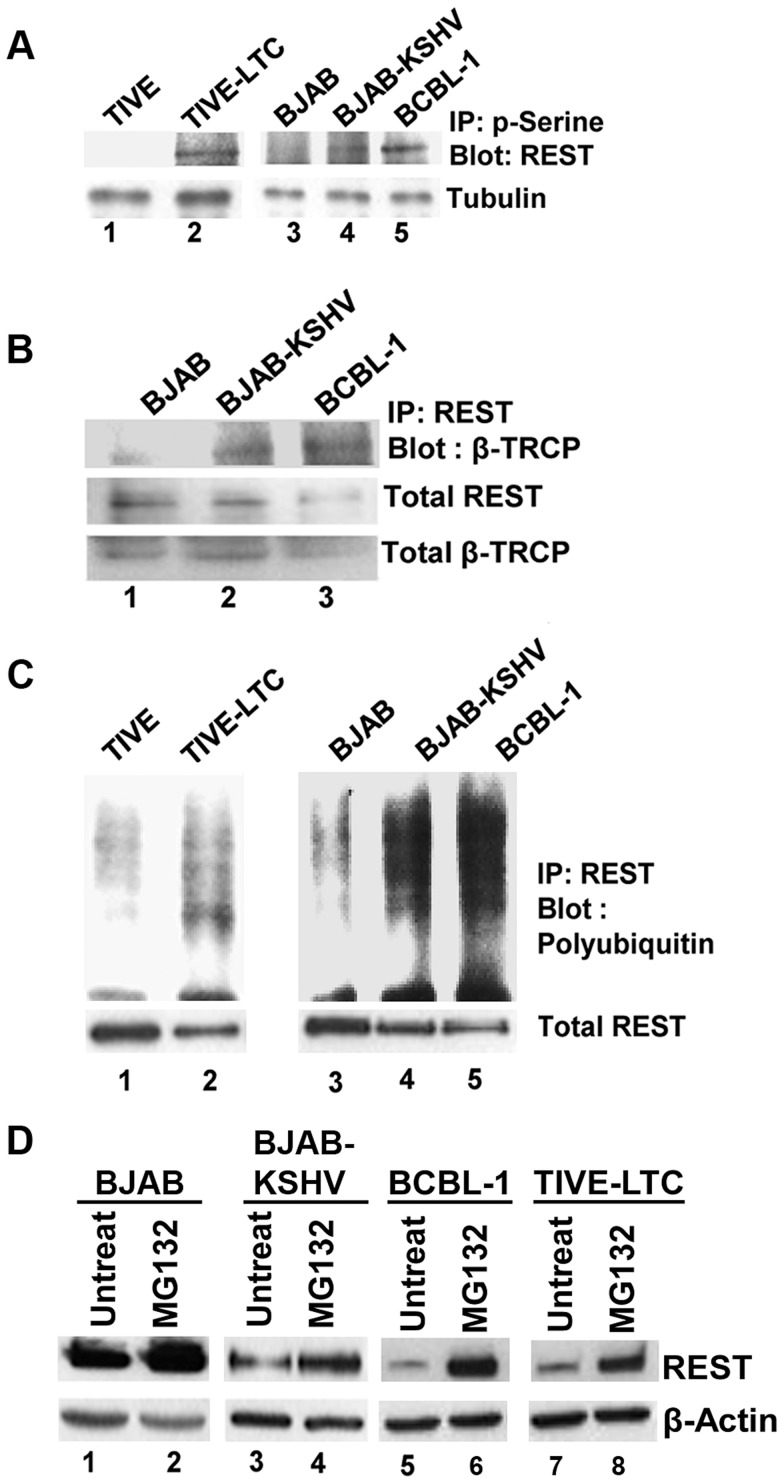
Phosphorylation and ubiquitination of REST in the cytoplasm of KSHV infected cells. A) Immunoprecipitation of cytoplasmic fractions from TIVE and TIVE-LTC, BJAB, BJAB-KSHV and BCBL-1 cells with anti-phosphoserine antibodies and analysis by immunoblotting with anti-REST antibody. B) Immunoprecipitation of the cytoplasmic fractions from BJAB, BJAB-KSHV and BCBL-1 cells with anti-REST antibody followed by immunoblot with anti-β-TRCP antibody. Bottom panel shows whole cell extracts Western blotted with anti-REST and β-TRCP antibodies. (C) Immunoprecipitation of cytoplasmic fractions from TIVE and TIVE-LTC, BJAB, BJAB-KSHV and BCBL-1 cells with anti-REST antibody followed by anti-ubiquitin immunoblotting. Bottom panel shows whole cell extracts subjected to Western blot using anti-REST antibody. (D) Effects of MG132 treatment on REST protein levels. BJAB, BJAB-KSHV, BCBL-1 and TIVE-LTC cells were treated with 10 µM MG132 for 6 h, and the cell lysates were Western blotted using REST antibody. As a control for loading, equivalent amount of protein samples were Western blotted with an anti-actin antibody.

It has been reported that serine phosphorylation of REST in the conserved phosphodegron motif promotes recognition by the E3 ubiquitin ligase β-TRCP and ubiquitination [40], [41]. As our data suggested an unexpected decrease of REST in the infected cells, we next asked whether the phosphorylation of REST in the cytoplasm was followed by its phosphorylation-dependent ubiquitination. To examine this regulatory role, we first verified the serine phosphorylation of REST in the cytoplasm of infected cells by immunoprecipitating with phosphoserine antibody and Western blotting with REST antibody. Consistent with the Western blot results (Figures 5C and D), a significant level of serine phosphorylation of REST was detected in KSHV-infected TIVE-LTC, BJAB-KSHV and BCBL-1 cells compared to a very low level of phosphorylation in KSHV-negative TIVE and BJAB cells (Figure 6A).

We next determined whether β-TRCP could be associated with phosphorylated REST in the cytoplasm of infected cells. Immunoprecipitation of cytoplasmic extracts of BJAB, BJAB-KSHV and BCBL-1 cells with REST and Western blots with anti-β-TRCP antibodies showed increased interaction of REST with β-TRCP in the infected cells, whereas it was barely detectable in uninfected cells (Figure 6B). We next determined whether REST degradation occurs in the cytoplasm of KSHV-infected cells. Analysis of cytosolic fractions from TIVE-LTC, BJAB-KSHV and BCBL-1 cells by immunoprecipitation with REST and Western blots for polyubiquitin revealed a higher level of ubiquitination in TIVE-LTC, BJAB-KSHV and BCBL-1 cells compared with TIVE and BJAB cells displaying lower levels of ubiquitination (Figure 6C). Thus, the ubiquitination levels of REST correlated with REST phosphorylation and the association of REST with β-TRCP in the cytoplasm.

In order to confirm that the ubiquitin proteasome system is involved in the degradation of REST in KSHV infected cells, BCBL-1 and TIVE-LTC cells were treated with the proteasome inhibitor MG132, and the cell lysates were Western blotted for REST. As shown in figure 6D, compared to the untreated cells (lanes 3, 5, and 7), MG132 treatment increased the protein level of REST in the infected BJAB-KSHV, BCBL-1, and TIVE-LTC cells (lanes 4, 6, and 8). However, MG132 treatment had no significant effect on the REST protein level in uninfected BJAB cells (lanes 1 and 2). This result further supported our finding that the degradation of REST observed in the infected cells was probably mediated by the ubiquitin proteasome pathway.

### KSHV latent protein Kaposin A mediates mGluR1 expression by REST relocalization

**Figure 7:**
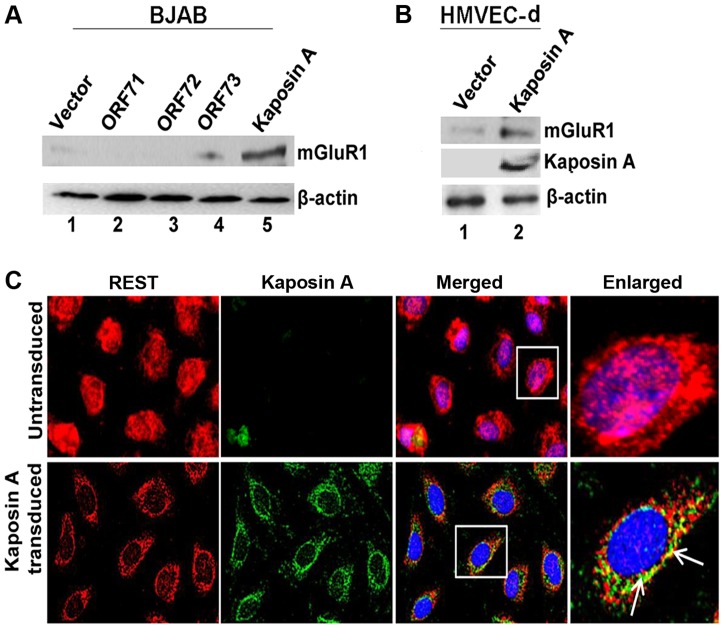
KSHV latency associated Kaposin A protein mediates mGluR1 expression. A) Kaposin A (ORFK12) over expression leads to mGluR1 expression in BJAB cells. BJAB cells were transduced with control lentivirus vector or lentiviruses expressing ORFs 71, -72, -73 and Kaposin A for 72 h. The expression levels of mGluR1 in the cell lysates were determined by Western blot using anti-mGluR1 antibody. B) mGluR1 and Kaposin A expression determined by Western blot in primary HMVEC-d cells following transduction with lentivirus control or lentivirus-Kaposin A. β-actin was used as loading control. C) Colocalization of REST and Kaposin A in HMVEC-d cells transduced with lentivirus vector, or lentivirus-Kaposin A after 72 h. Boxed areas are enlarged in the right panel. Arrows indicate colocalization of REST and Kaposin A. Magnifications 40×.

Since REST was more localized in the cytoplasm of latently infected cells, we hypothesized that latent KSHV protein(s) in the infected cells binds and sequesters REST in the cytoplasm, which in turn leads to overexpression of the mGluR1 gene. To determine the identity of the KSHV latent protein responsible for this, BJAB cells were transduced with the lentiviral constructs of KSHV latent ORF71, -72, -73, and Kaposin A genes, expression levels assessed by real-time PCR (Figure S4B), and mGluR1 level analyzed by Western blot. ORF K12 or Kaposin A transduction led to a robust increase in mGluR1 expression in BJAB cells, indicating the involvement of Kaposin A in the regulation of mGluR1 expression, whereas the other latent genes did not significantly induce the expression of mGluR1 (Figure 7A). mGluR1 expression in Kaposin A transduced BJAB cells was further confirmed by immunoprecipitation experiments (Figure S4C). Transduction efficiencies were determined by control lentiviral GFP expression (Figure S4D). We also observed higher levels of mGluR1 protein expression in primary HMVEC-d cells transduced with Kaposin A which further demonstrated the Kaposin A dependency of mGluR1 expression (Figure 7B).

To determine whether Kaposin A is responsible for the observed cytoplasmic relocalization of REST in the infected cells, we transduced HMVEC-d cells with a lentiviral Kaposin A construct (ORF K12) and localization was determined by IFA using anti-Kaposin A antibodies. This analysis revealed that a major portion of endogenous REST was translocated into the cytoplasm and colocalized with Kaposin A in the transduced cells (Figure 7C).

### Kaposin A interacts with REST in the cytoplasm of infected cells

**Figure 8:**
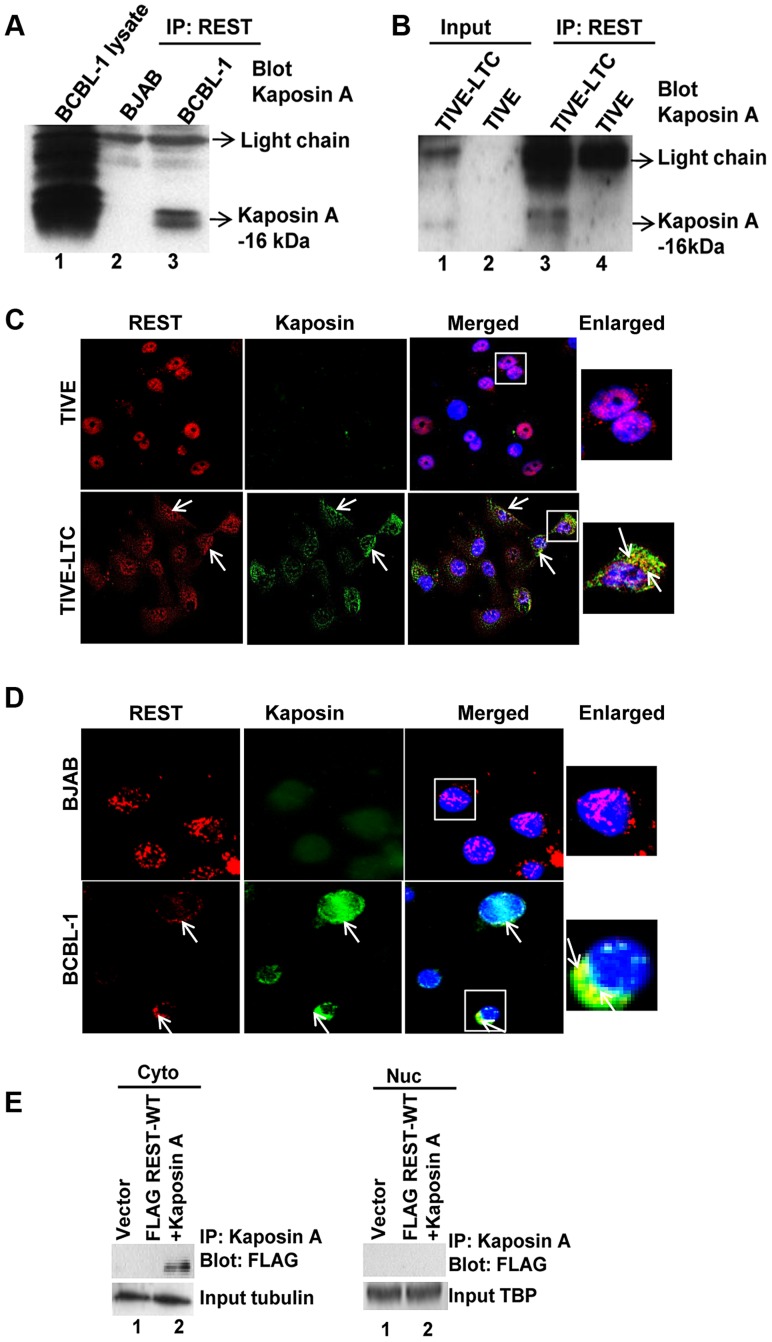
KSHV latency associated Kaposin A protein interacts with REST in the cytoplasm. (A and B) Immunoprecipitation of cytoplasmic fractions from BJAB and BCBL-1 cells (A), TIVE and TIVE-LTC cells (B) with anti-REST antibody followed by anti-Kaposin A immunoblotting. BCBL whole cell lysates were used as a positive control for Kaposin A expression. C) Colocalization of REST and Kaposin A in the cytoplasm of BJAB and BCBL-1 cells determined by immunostaining with anti-REST and anti-Kaposin A antibodies. D) Colocalization of REST and Kaposin in TIVE and TIVE-LTC cells. Nuclei were stained with DAPI. Arrows indicate colocalization of REST with Kaposin A. Boxed areas are enlarged on the rightmost panel. E) HEK 293T cells were transduced with vector alone or with FLAG-WT-REST and Kaposin A for 72 h. FLAG-tagged REST interaction with Kaposin A in the cytoplasm and nuclei was assessed by immunoprecipitation with Kaposin A antibody followed by immunoblotting with FLAG antibody.

To verify that REST binds to Kaposin A in the cytoplasm of KSHV infected cells, we immunoprecipitated REST from cytoplasmic fractions of both uninfected BJAB and KSHV-infected BCBL-1 cells and then Western blotted with anti-Kaposin A antibodies, which detected specific bands of Kaposin A at approximately 16–18 kDa (Figure 8A). BCBL-1 cell lysates used as positive control also identified 16–18-kDa immunoreactive bands of Kaposin A in the infected cells (Figure 8A). The predicted molecular weight of Kaposin A is 6-kDa; however, WB analyses often detect specific bands of about 16–18-kDa and above [42], [43], [44]. Similar immunoprecipitation analysis using TIVE and TIVE-LTC cells also revealed that REST interacts with Kaposin A in the infected cell cytoplasm (Figure 8B). We also observed the colocalization of REST and Kaposin A in the cytoplasm of TIVE-LTC (Figure 8C) and BCBL-1 cells (Figure 8D).

To further verify the physical interaction of REST with Kaposin A, we co-transduced 293T cells with Kaposin A and retroviral FLAG tagged REST and the cytoplasmic and nuclear lysates were immunoprecipitated with Kaposin A and Western blotted with anti-FLAG antibodies. This co-immunoprecipitation experiment demonstrated the ability of Kaposin A to interact with REST in the cytoplasm, but not in the nucleus (Figure 8E).

### Kaposin A colocalizes with REST in tissue samples of KS and PEL patients

**Figure 9:**
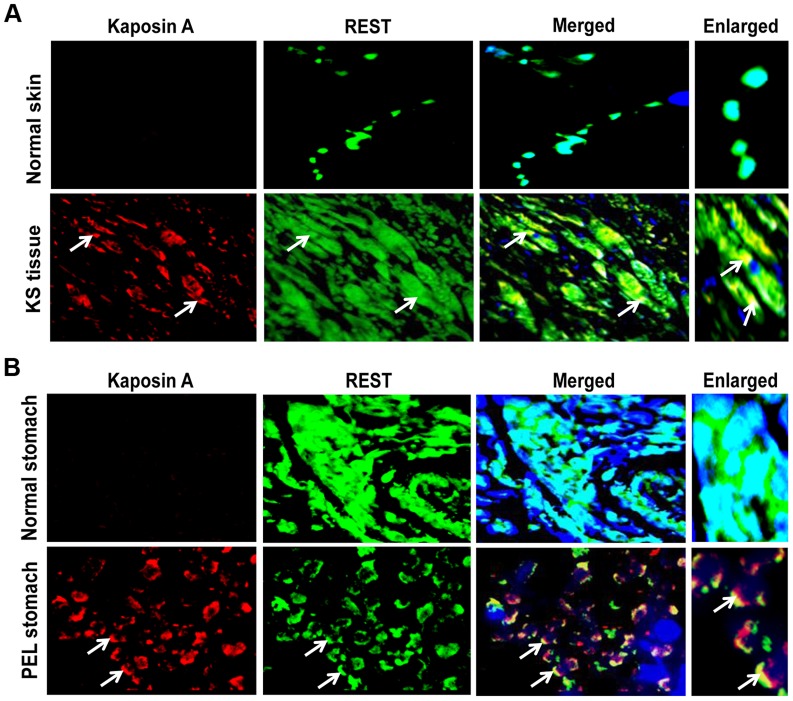
Colocalization of REST and Kaposin in KSHV-infected patient samples. (A) Immunofluorescence analysis of REST and Kaposin staining in normal human skin and KS tissue. (B) Immunofluorescence analysis of REST and Kaposin staining in normal stomach tissue and PEL affected stomach tissue. The tissue sections were counterstained with DAPI. White arrows indicate cytoplasmic localization of REST and Kaposin A, and their colocalization in KS and PEL tissues. Magnifications 80×. Boxed areas are enlarged.

We next examined the staining pattern and colocalization of REST and Kaposin A in KS and PEL patient samples and in normal tissues by immunofluorescence analysis. As shown in Figures 9A and B, strong nuclear staining of REST was observed in normal skin tissues as well as in normal stomach tissues. In contrast, cytoplasmic localization of REST and notable colocalization with Kaposin A were observed in the endothelial cells of KS as well as in the cells of PEL tissues, presumably the B cells. Together, these results suggested that Kaposin A expression regulates mGluR1 expression through interaction with REST in the cytoplasm of KSHV infected cells.

### Kaposin A regulates the phosphorylation of REST and interaction with β-TRCP for ubiquitination

As shown in figure 6B and C, phosphorylated REST interacts with β-TRCP and promotes the ubiquitination and degradation of REST in the cytoplasm of infected cells. It has previously been reported that REST has a degron motif and the phosphorylation of REST at serine 1024, 1027, and 1030 of the degron motif is required for the interaction of REST with β-TRCP during oncogenic transformation [41]. Because Kaposin A is a protein involved in transformation of infected cells [45], [46], we postulated that Kaposin A binding with REST phosphorylates REST at the 1024, 1027, and 1030 residues, leading to the interaction with β-TRCP and ubiquitination of REST.

**Figure 10:**
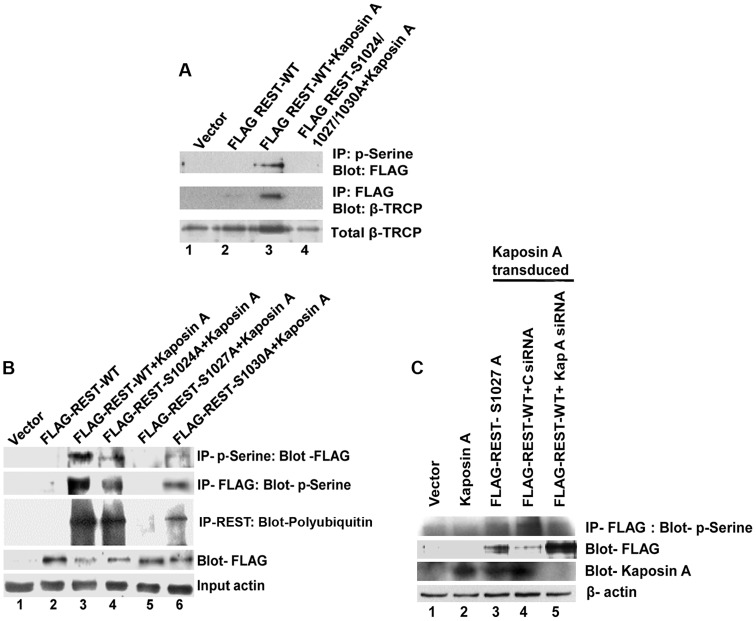
Effect of Kaposin A on phosphorylation of degron mutant REST. (A) 293T cells were transduced with vector alone or retroviruses expressing FLAG-WT-REST or Kaposin A and FLAG-WT-REST or Kaposin A and FLAG-mutant REST. Upper panel shows cytoplasmic fractions immunoprecipitated with anti-phosphoserine antibody followed by Western blot with anti-FLAG antibody. Middle panel shows cytoplasmic fractions immunoprecipitated with anti-FLAG antibody followed by Western blot with anti-β-TRCP. Bottom panel shows whole cell extracts subjected to Western blot using anti-β-TRCP antibody. (B) 293T cells were transduced with vector alone or Kaposin A for 2 days and the Kaposin A transduced cells were transfected with FLAG tagged REST-WT or FLAG-REST individually mutated at serine 1024, (FLAG-REST S1024A), 1027 (FLAG-REST S1027A), or 1030 (FLAG-REST S1030A) for 48 h. Cell lysates were IPed with anti-phosphoserine antibody followed by immunoblot with anti-FLAG antibody (First panel) or IP-ed with anti-FLAG antibody followed by immunoblot with anti-phosphoserine antibody (Second panel) or immunoprecipitated using anti-FLAG antibody and Western blotted with poly-ubiquitin antibody (Third panel). The fourth and fifth panels show cell lysates subjected to Western blot using anti-FLAG, and anti-β-actin, respectively. (C) Effect of Kaposin A siRNA on REST phosphorylation: 293T cells transduced with vector alone or Kaposin A were transfected with FLAG-REST S1027A or FLAG-REST WT followed by transfection with control siRNA (C siRNA) or a pool of Kaposin A specific siRNA (Kap A siRNA). After 48 h post-transfection, cell lysates were prepared and immunoprecipitated with anti- FLAG antibody followed by immunoblot with anti-phosphoserine antibody (First panel). The membrane was striped and reprobed with anti-FLAG antibody as shown in the second panel. Whole cell extracts were subjected to Western blots analysis using anti-Kaposin A for Kaposin A expression (Third panel). β-actin was used as loading control.

To investigate this, we co-transduced 293T cells with Kaposin A and FLAG REST-WT or FLAG-REST triple mutant (where all three phosphodegron residues are mutated-FLAG-REST-S1024/1027/1030A), cytoplasmic fractions immunoprecipitated with anti-phosphoserine antibodies and Western blotted with anti-FLAG antibodies. A significant level of serine phosphorylated REST was detected in Kaposin A and FLAG REST-WT transduced cells (Figure 10A, upper panel). In contrast, the serine phosphorylation of REST was severely impaired in Kaposin A and FLAG-REST triple mutant transduced cells suggesting that Kaposin A mediates REST phosphorylation in its phosphodegron sites. As Kaposin A is involved in REST phosphorylation in the conserved degron sites, we next determined whether phosphorylated REST binds to endogenous β-TRCP. As shown in Figure 10A, middle panel, immunoprecipitation with FLAG and Western blot with β-TRCP showed a markedly increased interaction of REST with endogenous β-TRCP in Kaposin A and FLAG REST-WT transduced cells, whereas no interaction was observed in Kaposin A and FLAG-REST triple mutant transduced cells. These data demonstrated that blocking Kaposin A mediated phosphorylation of REST weakens its association with endogenous β-TRCP.

To further investigate which specific degron site is phosphorylated by Kaposin A, we transiently transduced 293T cells with lentiviral Kaposin A and 48 h after transduction, the cells were transfected with pCMV-FLAG-REST WT plasmid or pCMV-FLAG-REST individually mutated at serine 1024, (pCMV-FLAG-REST-S1024A), 1027 (pCMV-FLAG-REST-S1027A), or 1030 (pCMV-FLAG-REST-S1030A). Cell lysates were immunoprecipitated using anti-phosphoserine antibodies followed by Western blotting with anti-FLAG antibodies or vice versa. As shown in Figure 10B, the serine 1027 mutant completely abolished the capacity for phosphorylation (Figure 10B, lane 5, first and second panel). However, the serine 1024 and 1030 mutants had no effect on phosphorylation compared to wild type REST (Figure 10B, lane 4 and 6, first and second panel), indicating that the phosphorylation of these two sites are not directly mediated by Kaposin A. The defect in REST phosphorylation in mutant serine 1027 suggested that Kaposin A initially phosphorylates REST on serine 1027. Phosphorylation on 1027 may provide the signal to phosphorylate the other degron residues.

In order to determine whether the serine 1027 induced phosphorylation is responsible for REST ubiquitination, the cell lysates immunoprecipitated with anti-FLAG antibody were analyzed by Western blotting with an anti-polyubiquitin antibody. Consistent with the increased phosphorylation, the ubiquitination was markedly increased in wild type REST, as well as in serine 1024 and 1030 mutants transfected cells (Figure 10B, lanes 3, 4, and 6, third panel). In contrast, the phosphorylation defective mutant 1027 failed to induce ubiquitination (Figure 10B, lane 5, third panel). These results suggest that serine 1027 mediated phosphorylation is required for the ubiquitination of REST. We also observed that the phosphorylation defective mutant FLAG-REST-S1027A stabilized REST (Figure 10B, lane 5, fourth panel). The observed reduction of REST in FLAG-REST WT and FLAG-REST-S1024A and -S1030A (Figure 10B, lanes 3, 4 and 6, fourth panel), after Kaposin A stimulation may be due to the degradation of phosphorylated REST at the 1027 residue. Taken together, our studies demonstrated that Kaposin A regulates REST phosphorylation in the conserved phosphodegron motif which enhances the ubiquitination of REST and thus reduces the level of REST.

To further verify the role of Kaposin A in REST phosphorylation, 293T cells transduced with vector alone or Kaposin A were transfected with FLAG-REST S1027 or FLAG-REST WT first and then transfected with control or Kaposin A specific siRNA. After 48 h post transfection, levels of REST phosphorylation were assessed by immunoprecipitating with anti-FLAG antibody followed by Western blotting with anti-phosphoserine antibody. We observed that compared to control siRNA transfected cells, Kaposin A siRNA transfected cells abolished the phosphorylation and degradation of REST in REST WT transfected cells (Figure 10C, lane 4 and 5, first and second panel). Kaposin A specific siRNA efficiently knocked down the expression of Kaposin A in the transduced cells (Figure 10C, lane 5, third panel). As expected, cells transfected with phosphorylation defective mutant REST-S1027 had no effect on phosphorylation (Figure 10C, lane 3, first panel). These data confirm that Kaposin A is essential for the phosphorylation of REST.

### mGluR1 antagonists and glutamate release inhibitor block proliferation of KSHV infected cells

**Figure 11:**
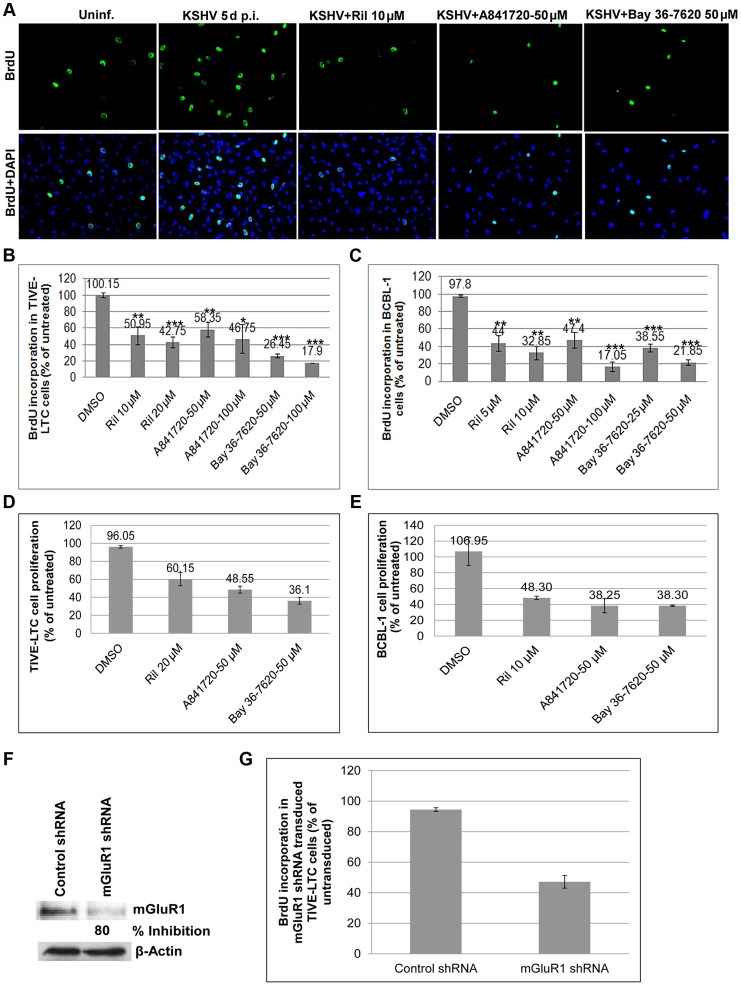
Glutamate release inhibitor and mGluR1 antagonists efficiently blocked KSHV infected cell proliferation. A) HMVEC-d cells were left uninfected or infected with KSHV for 3 d. The cells were then cultured in the absence or presence of riluzole (10 µM), A841720 (50 µM) or Bay36-7620 (50 µM) for 48 h followed by BrdU pulse labeling for 2 h. BrdU incorporation was detected by staining with a BrdU antibody. Bottom panel shows merged images of BrdU and DAPI. (B and C) BrdU incorporation determined by ELISA in TIVE-LTC (B), and BCBL-1 cells (C) treated in the absence or presence of different concentrations of riluzole, A841720, or Bay36-7620 for 48 h followed by BrdU pulse labeling for 2 h. BrdU incorporation was analyzed by a BrdU cell proliferation ELISA kit. Error bars represent the mean ± SD of three independent experiments. **p<0.001, ***p<0.0001 compared with DMSO treatment. (D and E). MTT assay for cell proliferation. TIVE-LTC (D) and BCBL-1 cells (E) were incubated with riluzole, A841720, or Bay36-7620 for 48 h. At the end of incubation cell growth was measured with Vibrant MTT cell proliferation assay kit as described in materials and methods. Data are mean ± SD. (F and G) mGluR1 shRNA transduction: TIVE-LTC cells transduced with control shRNA lentivirus or with mGluR1 shRNA lentivirus were selected using puromycin hydrochloride. Cell lysates Western blotted with anti mGluR1 antibody showed 80% knockdown of mGluR1 expression in mGluR1 shRNA transduced cells. β-actin was used as a loading control. (G) BrdU incorporation determined by a BrdU cell proliferation ELISA in control and mGluR1 shRNA transduced TIVE-LTC cells. Error bars represent the mean ± SD.

We next focused on the biological response of glutamate release and binding to its receptors. We postulated that the glutamate released by infected cells binds to mGluR1 permitting cellular signaling and the proliferation of glutamate secreting infected cells. To determine the effects of glutamate and mGluR1 on cell proliferation, primary HMVEC-d cells infected with KSHV for 3 d were cultured for 2 d in the presence or absence of glutamate release inhibitor riluzole, and mGluR1 antagonists A841720 and Bay 36-7620, pulsed with BrdU for 2 h and BrdU incorporation determined by IFA. As shown in Figure 11A, HMVEC-d cells infected with KSHV for 5 d showed a much higher rate of proliferation than the uninfected cells. This increased proliferation of HMVEC-d cells was significantly reduced by exposure to riluzole, A841720 and Bay 36-7620 (Figure 11A). These results were also confirmed by BrdU cell proliferation ELISA (Figures S5A and B).

We further tested the involvement of riluzole, A841720, and Bay 36-7620 in TIVE and TIVE-LTC, BJAB and BCBL-1 cell proliferation by BrdU cell proliferation ELISA. No treatment and vehicle treatment were used as controls. As shown in Figure 11B, treatment with riluzole, A841720 and Bay 36-7620 showed a concentration dependent decrease in the proliferation of both TIVE-LTC and BCBL-1 cells (Figures 11B and C). Due to the absence or low level of expression of mGluR1 receptors, only a minimal effect was observed in the proliferation of uninfected TIVE and BJAB cells (Figures S5C and D). To further confirm the effect of inhibitors on cell proliferation, cells treated with riluzole, A841720 or Bay 36-7620 were monitored using a vibrant MTT cell proliferation assay kit. Riluzole, A841720, and Bay 36-7620 caused a 60–70% decrease in cell growth compared to the untreated control (Fig. 11 D and E). Next, we confirmed the role of mGluR1 on the proliferation of infected cells by using mGluR1 shRNA. TIVE- LTC cells transduced with mGluR1 shRNA or control shRNA were assayed for BrdU incorporation. Compared to control shRNA cells, mGluR1-shRNA significantly reduced the proliferation of TIVE-LTC cells (Fig. 11G), indicating that mGluR1 plays a key role in the proliferation of KSHV infected cells.

Collectively, these results suggested that riluzole and mGluR1 antagonists suppressed the binding of glutamate to the receptors of infected cells and thereby arresting the activation of receptors by glutamate leading into the proliferation of KSHV infected cells.

## Discussion

Glutamate release along with autocrine and paracrine glutamate receptor signaling has been demonstrated to accelerate cell proliferation and tumor progression [47], [48]. During the latent phase of KSHV infection, the cytokines and growth factors released into the extracellular milieu play significant roles in the long term proliferation, survival, and maintenance of the infected cells which probably results in KSHV associated malignancies [8], [12], [13], [15], [16], [17], [18], [49]. Our comprehensive studies demonstrating the increased secretion of glutamate into the cytokine milieu in response to KSHV infection suggest that glutamate could be acting as an autocrine and paracrine growth factor during KSHV induced oncogenesis. Secretion of glutamate occurs in uninfected and infected cells, with comparatively low levels in uninfected cells. We have demonstrated that KSHV infection and appropriate viral gene expression are critical for the generation and release of glutamate in the infected cells. As the viral genome persists in a latent state in the infected cells, the expression level of the latent genes may affect glutamate secretion. Our current study clearly suggested a mechanism whereby the latent ORF73 gene expression affect the stability of c-Myc activation and the depletion of which resulted in reduced glutaminase expression and glutamate secretion. This implies that the level of infection and consistent expression of viral genes are required for the continued secretion of glutamate.

Our studies also show that KSHV infected cells induced the highest levels of glutaminase expression and caused a moderate increase in glutamate release. This difference could be attributable to glutamate transporters and the uptake of glutamate into cells. The glutamate taken up by the cells is converted into glutamine via the glutamine synthetase pathway [50]. Since there are several evidences to indicate that glutamate uptake and its enzymatic conversion are significant steps to maintain extracellular glutamate concentration [50], [51], [52], it is possible that expression or functional impairment of glutamate transporters may also be involved in the maintenance of extracellular glutamate levels in the infected cells.

c-Myc has numerous significant effects on cancer cell metabolism by modifying expression of proteins involved in metabolic pathways [53]. It is known to stimulate increased expression of its target proteins and glutaminase expression by transcriptional repression of mir23a/b in cancer cells [34]. The increased c-Myc activity may also significantly alter the metabolism of glutamine in the infected cells. These changes in glutamine metabolism may profoundly influence the synthesis of molecules involved in growth and survival of infected cells. Although increased glutaminolysis is a supplementary source of energy and may provide significant benefits in terms of the survival of the infected cells, they require additional factors for the induction of cell proliferation or transformation. Thus, while the role of increased metabolism and the components involved in metabolism remains to be determined, it is clear from our study that the secreted glutamate is being used to activate mGluR1 which contribute to the proliferation of infected cells.

**Figure 12:**
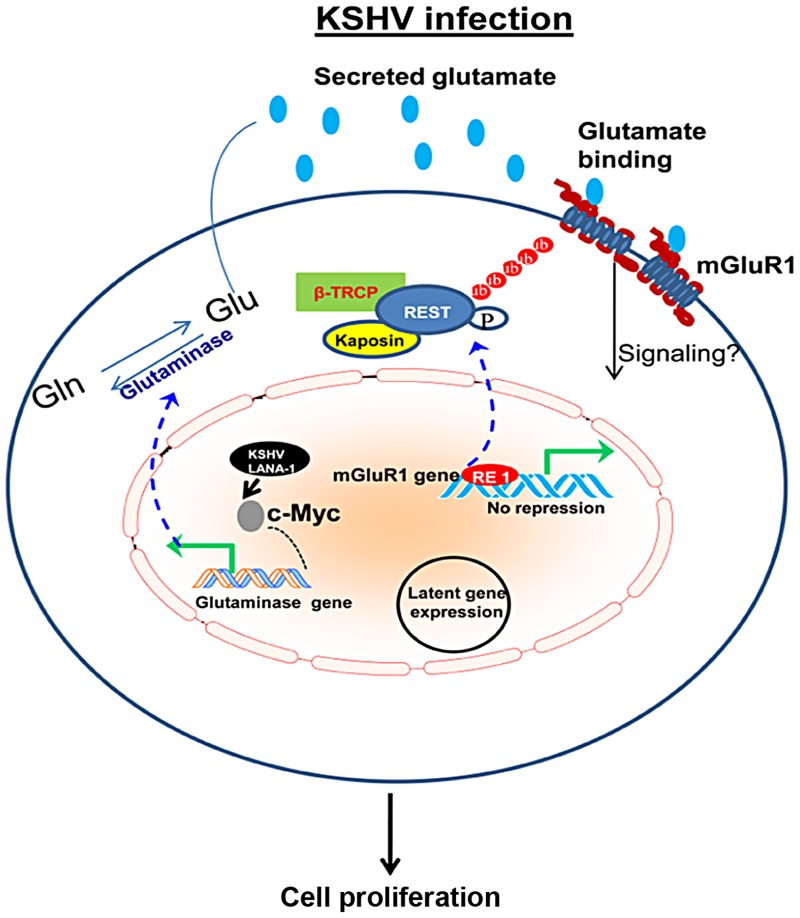
Schematic diagram showing the pathways for glutamate generation and mGluR1 upregulation in KSHV infected cells. During latent infection, KSHV LANA-1 protein activates c-Myc, which leads to the upregulation of glutaminase and induction of glutamate release. Dashed line denotes c-Myc regulation and glutaminase expression. KSHV latent viral protein Kaposin A binds and sequesters REST in the cell cytoplasm, which in turn relieves the REST mediated suppression of the mGluR1 gene and upregulates expression of the mGluR1 receptor. Binding of glutamate to mGluR1 induces signaling and proliferation of infected cells. These studies show that proliferation of cancer cells latently infected with KSHV in part depends upon glutamate and glutamate receptor and therefore could potentially be used as therapeutic targets for the control and elimination of KSHV associated cancers.

Interestingly, we report that KSHV infected cells also upregulate the expression of glutamate receptor mGluR1, which in turn results in increased proliferation as a result of glutamate binding to mGluR1 in the infected cells. Enhanced expression of mGluR1, and the intracellular signaling pathway activated by mGluR1, has the ability to induce cell proliferation and oncogenic transformation [35], [36]. Our data provide evidence that mGluR1 is upregulated in *in vitro* latently infected cells and *in vivo* patient samples. Mechanistically, mGluR1 overexpression involves relocalization of REST from the nucleus to the cytoplasm and loss of REST expression in the infected cells. Decreased REST expression, relocalization of REST, and degradation of REST are possible adaptations to antagonize REST-mediated effects to accomplish the overexpression of mGluR1 [39], [54], [55]. A remarkable difference in the pattern of REST localization observed in the infected cells indicates that mGluR1 expression may be regulated via the relocalization of REST. Translocation of REST to the cytoplasm relieves the NRSE or RE1 mediated transcriptional repression in the promoter regions of mGluR1 and upregulates its transcription (Figure 12). Another one of our major findings is that the KSHV latent protein Kaposin A is responsible for cytoplasmic relocalization of REST and mGluR1 activation (Figure 12). Kaposin A mediated oncogenesis has been demonstrated *in vitro* in Rat3 fibroblasts and in nude mice [45], [46]. Previous studies have suggested that Kaposin A regulates oncogenesis by influencing the phosphorylation of signaling molecules involved in cellular processes, such as cell proliferation and gene transcription [42], [46]. Our findings suggest that sequestration of REST in the cytoplasm by Kaposin A modulates phosphorylation-dependent ubiquitination of REST by altering the phosphorylation status of REST (Figure 12). Kaposin A regulates REST phosphorylation at the specific degron sites which are essential for binding to β-TRCP and degradation of REST during oncogenic transformation. Thus, the downregulation of REST, which is seen in actively proliferating cancer cells [56], [57], might be involved in the regulation of mGluR1 and in cellular transformation during KSHV induced cancer development.

Since Kaposin A does not have a known protein kinase domain, how Kaposin A binding to REST induces phosphorylation of REST needs to be elucidated. Several mechanisms are possible to account for the phosphorylation of REST by Kaposin A. Kaposin A has been reported to phosphorylate a number of kinases involved in cell proliferation [46]. Therefore, it is possible that Kaposin A may couple through one of these kinases for the activation of REST and recruitment of β-TRCP. It is also possible that the interaction of Kaposin A with REST may induce the phosphorylation of REST by allowing a conformational change. These modifications would create a favorable molecular environment for the cross talk between REST and β-TRCP.

In addition to Kaposin A mediated mGluR1 expression, we observed that over expression of LANA-1 also lead to a slight increase in mGluR1 expression. This increase in mGluR1 expression may be a result of an alteration in the N-terminal repressor domain of REST on the mGluR1 promoter. The N-terminal repression domain of REST represses target gene expression by recruiting the transcriptional corepressor mSin3 and then forming a complex with histone deacetylase (HDAC) [58], [59]. Since LANA-1 has already been shown to be associated with mSin3 co-repressor as well as with HDAC [60], it is expected that LANA-1 may be able to bypass REST mediated repression by sequestration of the mSin3/HDAC complex which results in the expression of mGluR1 genes. It is also known that mSin3/HDAC regulated repression is not sufficient for complete transcriptional repression of REST target genes [58], [59], [61]. Therefore, the slight induction of mGluR1 expression in LANA-1 expressing cells could be due to the partial derepression of REST target genes by LANA-1.

Glutamate receptor antagonists and glutamate release inhibitors were shown to be effective in suppressing the proliferation of non-neuronal cancer cells [62], [63]. Identification of the activity of glutamate and mGluR1 in glioma and melanoma development has been the rational approach for testing glutamate release inhibitors talampanel and riluzole in clinical trials for the treatment of glioma and melanoma, respectively [64], [65]. Our functional data shows that the increased production of glutamate and expression of mGluR1 in response to KSHV infection promotes the proliferation of infected cells. Several studies have demonstrated that the signaling pathways activated by mGluR1 contribute to the proliferation and survival of cancer cells [22], [66]. Further studies are essential to determine the role of glutamate and mGluR1 activity in signal induction, viral gene expression, and viral genome maintenance in cells latently infected with KSHV. The blocking effect of riluzole, and the mGluR1 antagonists on proliferation of KSHV infected cells suggests that these molecules could potentially be used for the treatment of KSHV associated malignancies by directly targeting the glutamatergic system in the infected cells.

## Materials and Methods

### Cell culture

Primary human dermal microvascular endothelial cells (HMVEC-d cells CC-2543) were purchased from Clonetics, Walkersville, MD. KSHV negative B-lymphoma cell line BJAB, and the KSHV latently infected B-cell line BCBL-1, were obtained from ATCC. BJAB-KSHV (KSHV–GFP recombinant virus in BJAB) was a gift from Dr. Blossom Damania (University of North Carolina, Chapel Hill). TIVE (telomerase-immortalized vein endothelial cell line) and TIVE LTC cells (TIVE cells carrying KSHV in a latent state) were a gift from Dr. Rolf Renne (University of Florida). These cell lines were maintained as described previously [67].

### Virus

Induction of the KSHV lytic cycle with TPA in BCBL-1 cells, and KSHV purification procedures have been previously described [68]. UV-treated replication-defective KSHV was prepared by exposing the purified virus stock to UV light (365 nm) for 20 min at a 10-cm distance. KSHV DNA was extracted from live KSHV and UV-treated KSHV, and the copies were quantitated by real-time DNA PCR using primers amplifying the KSHV ORF73 gene as described previously [7]. Unless stated otherwise, primary cells were infected with KSHV at 50 MOI (multiplicity of infection) per cell at 37°C.

### Antibodies and reagents

Rabbit anti-mGluR1 and β-TRCP antibodies as well as mouse anti-BrdU antibodies were from Cell Signaling, Beverly, MA. Mouse anti-glutaminase and rabbit anti-mGluR1 and -TATA binding protein (TBP) antibodies were from Abcam, Cambridge, MA. Mouse anti-tubulin and β-actin antibodies were from Sigma, St. Louis, MA. Mouse anti-c-Myc (9E10) and REST antibodies were from Santa Cruz, Santa Cruz, CA. Rat anti-Kaposin A/C and mouse anti-polyubiquitin antibodies were from Millipore, Temecula, CA. Mouse anti-ORF73 antibodies were generated in Dr. Chandran's laboratory. Anti-rabbit and anti-mouse antibodies linked to horseradish peroxidase were from KPL Inc., Gaithersburg, Md. Alexa 488 and 594 conjugated secondary antibodies were from Invitrogen. Protein A and G–Sepharose CL-4B beads were from Amersham Pharmacia Biotech, Piscataway, NJ. Lambda phosphatase (λPPase), and L-DON (6-diazo-5-oxo-norleucine) were from Santa Cruz. Riluzole, A841720 and Bay 36-7620 were from Tocris Bioscience, Minneapolis, MN.

### Plasmids

Plasmids encoding FLAG-tagged human REST wild-type and site-specific REST mutant plasmids (pCMV-FLAG-REST-S1024A, pCMV-FLAG-REST-S1027A, pCMV-FLAG-REST-S1030A), wild type FLAG-REST and triple mutant FLAG-REST-S1024/1027/1030A cloned into retroviral vector pQCXIN were provided by Dr. Stephen Elledge [41] (Harvard Medical School). Lentiviral constructs of KSHV ORF71 (vFLIP), ORF72 (vCyclinD), ORF73 (LANA-1) and ORFK12 (Kaposin A) were obtained from Dr. Chris Boshoff at the UCL Cancer Institute [69]. A plasmid encoding c-Myc shRNA sequence (plasmid #29435) was from Addgene. Transfection was performed using 5 µg of plasmid DNA and lipofectamine 2000 (Invitrogen) as per the manufacturer's instructions.

### Lentivirus production and transduction

Lentivirus was produced by transfection with a four-plasmid system, as previously described [70]. Briefly, 293T cells were transiently transfected with lentiviral constructs and the plasmid packaging system (Gag-Pol, Rev and VSV-G), the supernatants were collected, and filtered. Infections were carried out by incubating the virus preparation with cells in the presence of polybrene. The infection efficiency was estimated by analyzing GFP-expressing lentiviral vectors as positive controls. The expression levels of transduced viral genes were assessed by real-time PCR.

For mGluR1 knockdown, lentiviruses encoding mGluR1 shRNA or control shRNA were purchased from Santa Cruz Biotechnology. TIVE-LTC cells were transduced with control lentivirus shRNA and mGluR1 lentivirus shRNA according to the manufacturer's instructions and selected by puromycin hydrochloride.

### Quantification of glutamate release

An equal number of uninfected and infected cells were used for the experiments. Supernatants harvested at different times were centrifuged and glutamate levels were determined in 96-well plates by using a glutamate assay kit as per the manufacturer (Biovision, Mountain View, CA). The concentration of glutamate was determined by measuring the absorbance at 450 nm with a microplate reader.

### RT-PCR determination of mRNA expression

Total RNA was isolated with TRIzol Reagent (Invitrogen) and treated with DNase I (Ambion) at 37°C for 30 min. Reverse transcription was performed using a High-Capacity cDNA reverse transcription kit (Applied Biosystems). Regular PCR for mGluR1 was performed using 5 µl of the synthesized cDNA using appropriate forward and reverse primers *as* described by Choi et al [71]. PCR primers were as follows: mGluR1 5′-GTGGTTTGATGAGAAAGGAG-3′ (forward) and 5′-GTTGCTCCACTCAAGATAGC-3 (reverse). β-actin 5′-GCTCACCATGGATGATGATATCGCC-3′ (forward) and 5′GGATGCCTCTCTTGCTCTGGGCCTC-3′ (reverse).

Quantitative real time-PCR was performed with SYBR Green and an ABI prism 7000 sequence detection system (Applied Biosystems, Foster City, CA). The comparative Ct method was used to quantitate gene expression relative to the uninfected control. The following primer set was used: REST (forward 5′-GAGGAGGAGGGCTGTTTACC-3′; reverse 5′-TCACAGCAGCTGCCATTTAC-3′).

Primers used for qRT-PCR of viral genes: ORF71 (forward 5′-AGGTTAACGTTTCCCCTGTTAGC-3′; reverse, 5′-AGCAGGTCGCGCAAGAG-3′), ORF72 (forward-5′-AGCTGCGCCACGAAGCAGTCA-3′; reverse, 5′-CAGGTTCTCCCATCGACGA-3′), ORF73 (forward 5′-CGCGAATACCGCTATGTACTCA-3′; reverse 5′-GGAACGCGCCTCATACGA-3′), Kaposin A (forward 5′ GGATAGAGGCTTAACGGTGTTT-3′; reverse 5′-CAGACAAACGAGTGGTGGTATC-3′).

### siRNA transfection

A pool of two siRNAs synthesized by Integrated DNA technologies (IDT) were used to knockdown Kaposin A. siRNA sequences were as follows: siRNA1- 5′-r(UUGCAACUCGUGUCCUGAAUGCUACGG)-3′, siRNA2-5′- r(CCACAAACACCGUUAAGCCUCUAUCCA)-3′. Cells were transfected with siRNA at 100 pmol (50 pmol each) using siLentFect (Biorad) according to the manufacturer's instructions. Cell lysates were collected at 48 h post-siRNA transfection for immunoprecipitation and Western blot analysis.

### Immunofluorescence staining of tissue sections

Formalin-fixed, paraffin-embedded tissue samples from healthy subjects and patients with KS and primary effusion lymphoma were obtained from the ACSR (AIDS and Cancer Specimen Resource). Sections were deparaffinized with HistoChoice clearing reagent and rehydrated through ethanol to water. For antigen retrieval, the sections were microwaved in 1 mmol/l EDTA (pH 8.0) for 15 min, permeabilized with 0.5% Triton X-100 for 5 min, and then blocked with blocking solution (Image-iT FX signal enhancer-Invitrogen) for 20′ at RT. Immunostaining was performed using anti-mGluR1 and anti-mouse LANA-1 antibodies, followed by Alexa-488 and Alexa-594 conjugated secondary antibodies. Nuclei were stained with 4′,6-diamidino-2-phenylindole (DAPI) (Molecular Probes, Invitrogen), and stained cells were viewed under a fluorescence microscope with a 20× objective and the Nikon Metamorph digital imaging system.

### Immunocytochemistry

Cells grown on 8 well chamber slides (Nalge Nunc International) were fixed with 4% paraformaldehyde for 15 min, permeabilized with 0.2% Triton X-100, and blocked with Image-iT FX signal enhancer (Invitrogen) for 20 min. Cells were then incubated with primary antibodies against the specific proteins and subsequently stained with Alexa 488 or 594 conjugated secondary antibodies. Cells were mounted in mounting medium containing DAPI. Images were acquired using a Nikon 80i fluorescent microscope equipped with a Metamorph digital imaging system.

### Western blot

Cells were lysed in RIPA buffer containing 15 mM NaCl, 1 mM MgCl2, 1 mM MnCl2, 2 mM CaCl2, 2 mM phenylmethylsulfonyl fluoride, and protease inhibitor mixture (Sigma). The cell lysates were centrifuged at 13,000× *g* for 20 min at 4°C. Samples mixed with sample buffer containing β-mercaptoethanol, heated at 95°C for 5 min, and separated by SDS PAGE. The protein samples were then Western blotted with the indicated primary antibodies followed by incubation with species-specific HRP-conjugated secondary antibodies. Immunoreactive bands were visualized by enhanced chemiluminescence (Pierce, Rockford, IL) according to the manufacturer's instructions. To determine the fold change, blots were scanned, and quantified by densitometric analysis (Alpha Innotech Corporation, San Leonardo, CA) and normalized with respect to the amount of β-actin.

### Immunoprecipitation

For immunoprecipitations, 300–500 µg of cell lysates prepared in RIPA buffer or in NP-40 buffer were incubated with the appropriate primary antibody for 4–8 h with end-over-end rotation at 4°C, and the precipitated proteins captured by Protein A or G-Sepharose. The samples were Western blotted with specific primary and secondary antibodies.

### Proliferation assays

#### BrdU immunofluorescence analysis

Cells incubated with the inhibitors for 2 days were pulsed with BrdU (5-bromo-2′-deoxyuridine) for 2 h, and then fixed on the slides with 4% paraformaldehyde for 15 min, and permeabilized with 0.2% Triton X-100 for 5 min. The cells on slides were incubated in 1N HCl for 10 minutes at room temperature and blocked with Image-iT FX signal enhancer (Invitrogen). Cell proliferation was measured based on the incorporation of BrdU into chromosomal DNA by using anti-BrdU antibodies and Alexa-488 secondary antibodies.

#### BrdU ELISA

Various concentrations of glutamate release inhibitor and antagonists on uninfected and infected cell proliferation were determined by using a BrdU Cell Proliferation ELISA kit (Cell Signaling Technology). Briefly, uninfected and infected cells exposed to 5–20 µM riluzole (retreated every 12 h), A841720 (50–100 µM), and Bay 36-7620 (50–100 µM) were cultured for 2 days. After 2 days of treatment, cells were pulsed with BrdU for 2 h. The ELISA was performed in triplicate and the absorbance was read at 450 nM. BrdU incorporation was calculated as the percentage of BrdU incorporated with respect to the untreated control.

#### MTT cell proliferation assay

An equal number of cells plated in 96-well plates were treated with DMSO, riluzole, A841720 or Bay 36-7620 for 48 h. After the incubation, cell growth was monitored using the Vibrant MTT Cell Proliferation Assay Kit (Molecular Probes). Briefly, cells were incubated with MTT labeling reagent for 4 h, then the solubilization solution (sodium dodecyl sulfate (SDS) in HCl) for 4 h, and the absorbance was read at 570 nM with a microplate reader.

#### Nuclear and cytosol fractionation

Uninfected and infected cells were subjected to nuclear and cytosolic fractionation as described previously with slight modification [72]. Briefly, cells harvested in homogenization buffer (250 mM sucrose, 20 mM HEPES, 10 mM KCL, 1 mM EDTA, 1 mM EGTA and protease inhibitors) were subjected to centrifugation at 3,000 rpm for 5 min. The post-nuclear supernatant was centrifuged at 8,000 rpm for 5 min at 4°C. The supernatant was again centrifuged at 40,000 rpm for 1 h at 4°C, and the supernatant was collected as the cytosolic fraction. The nuclear pellet was lysed with NP-40 buffer and cleared at 13,000 rpm for 10 min. The purity of cytosolic and nuclear proteins was confirmed by immunoblotting with anti-tubulin and anti-TBP antibodies, respectively.

### Statistical analyses

Statistical significance was calculated using a two tailed Student's *t*-test. *P*<0.05 was considered significant.

## Supporting Information

Figure S1 A) L-DON treatments decreased glutamate release: BJAB, BJAB-KSHV, and BCBL-1 cells were left untreated or treated with L-DON (500 µM and 1 mM) for 24 h, and the collected supernatants were analysed for glutamate release. L-DON treatment at 500 µM and 1 mM showed ∼50% and 65% decreased secretion of glutamate, respectively, in BJAB-KSHV and BCBL-1 cells compared with untreated control. B) BJAB cells were transduced with control or ORF73 followed by transduction with c-Myc specific shRNA. After 48 hours of transduction, the media was replaced with fresh medium and cultured for a further 24 h; supernatants were collected and measured for the release of glutamate. Error bars represent the mean ± SD.
(TIF)

Figure S2 (A) HMVEC-d cells left uninfected or infected with KSHV for 5 d were immunoprecipitated using anti-mGluR1 antibodies and Western blotted with anti-mGluR1 antibody. (B) BJAB, BJAB-KSHV, BCBL-1, TIVE and TIVE-LTC cell lysates immunoprecipitated using anti-mGluR1 antibodies were Western blotted with anti-mGluR1 antibody. An equal amount of cell lysates were subjected to Western blot with β-actin used as loading control.
(TIF)

Figure S3 REST expression in TIVE, TIVE-LTC (A), BJAB, BJAB KSHV, and BCBL cells (B): Expression of REST mRNA assessed by quantitative RT–PCR and expressed as fold change determined using the comparative ct value based method. The fold change in gene expression is relative to the uninfected control equals 1. (C and D) Protein expression of REST in different cell lines was analyzed by immunoblotting. β-actin was used as an internal control. Percentage reduction is relative to the uninfected control.
(TIF)

Figure S4 (A) Cytoplasmic extracts from TIVE-LTC cells were either left untreated or treated with λ-phosphatase or with λ-phosphatase and phosphatase inhibitors then Western Blotted with REST. Tubulin was used as loading control. Lambda phosphatase treatment was done according to the manufacturer's instructions (Santa Cruz). (B) Viral gene expression in BJAB cells: BJAB cells were transduced with the lentiviral constructs of KSHV latent ORF71, -72, -73, and –Kaposin A genes. mRNA expression of viral genes were measured by real-time PCR analysis. Results were normalized to the amount of tubulin mRNA. C) Cell extracts from the control lentivirus vector or ORFK12 transduced cells or BCBL-1 cells as a positive control were subjected to immunoprecipitation using anti-mGluR1 antibody and Western blotted with mGluR1 antibody. β-actin was used as input loading control. D) Image showing lentiviral GFP expression in BJAB cells: BJAB cells were transduced with GFP expressing lentivirus for 3 d, and the transduction efficiency was determined by observing the GFP-positive cells under an immunofluorescence microscope.
(TIF)

Figure S5 BrdU incorporation determined by ELISA in uninfected (A) and KSHV infected (B) HMVEC-d cells. HMVEC-d cells seeded in 96 well plates were left uninfected or infected with KSHV for 3 d. The cells were then cultured in the absence or presence of riluzole (5 and 10 µM), A841720 (50 µM), or Bay36-7620 (50 µM) for 48 h followed by BrdU pulse labeling for 2 h. BrdU incorporation was quantitated using a BrdU cell proliferation ELISA kit. (C and D) BrdU incorporation determined by ELISA in TIVE (C) and BJAB cells (D) treated in the absence or presence of different concentrations of riluzole, A841720, or Bay36-7620 for 48 h followed by BrdU pulse labeling for 2 h. BrdU incorporation was analyzed by a BrdU cell proliferation ELISA kit.
(TIF)
