# Supplementary figures and images for: Glutamate Secretion and Metabotropic Glutamate Receptor 1 Expression during Kaposi's Sarcoma-Associated Herpesvirus Infection Promotes Cell Proliferation
Source: PLoS Pathog. 2014 Oct 9;10(10):e1004389. doi: 10.1371/journal.ppat.1004389 (PMC4192595; doi:10.1371/journal.ppat.1004389)

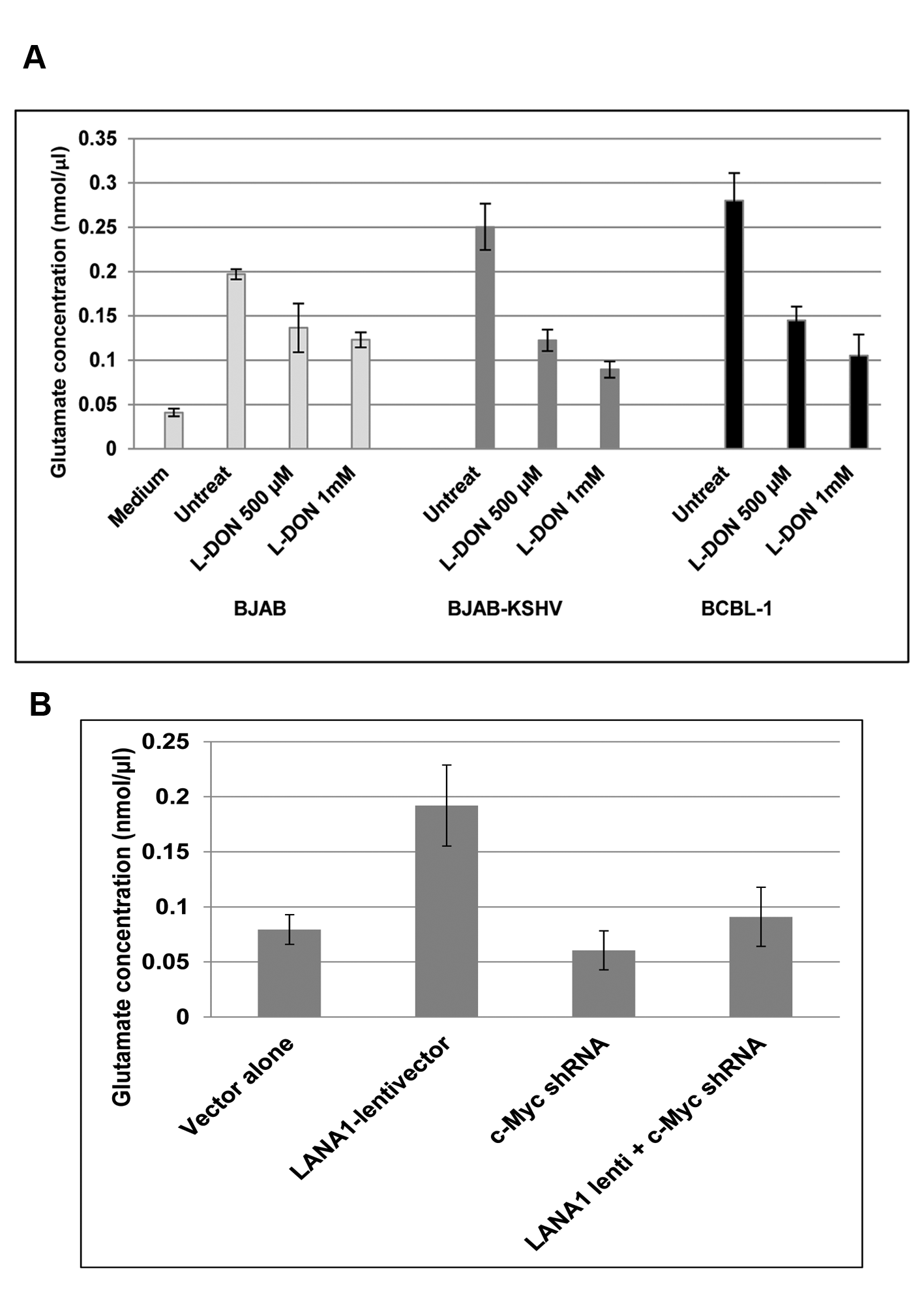

Supplement: Figure S1 — A) L-DON treatments decreased glutamate release: BJAB, BJAB-KSHV, and BCBL-1 cells were left untreated or treated with L-DON (500 µM and 1 mM) for 24 h, and the collected supernatants were analysed for glutamate release. L-DON treatment at 500 µM and 1 mM showed ∼50% and 65% decreased secretion of glutamate, respectively, in BJAB-KSHV and BCBL-1 cells compared with untreated control. B) BJAB cells were transduced with control or ORF73 followed by transduction with c-Myc specific shRNA. After 48 hours of transduction, the media was replaced with fresh medium and cultured for a further 24 h; supernatants were collected and measured for the release of glutamate. Error bars represent the mean ± SD. (TIF) [file ppat.1004389.s001.tif]

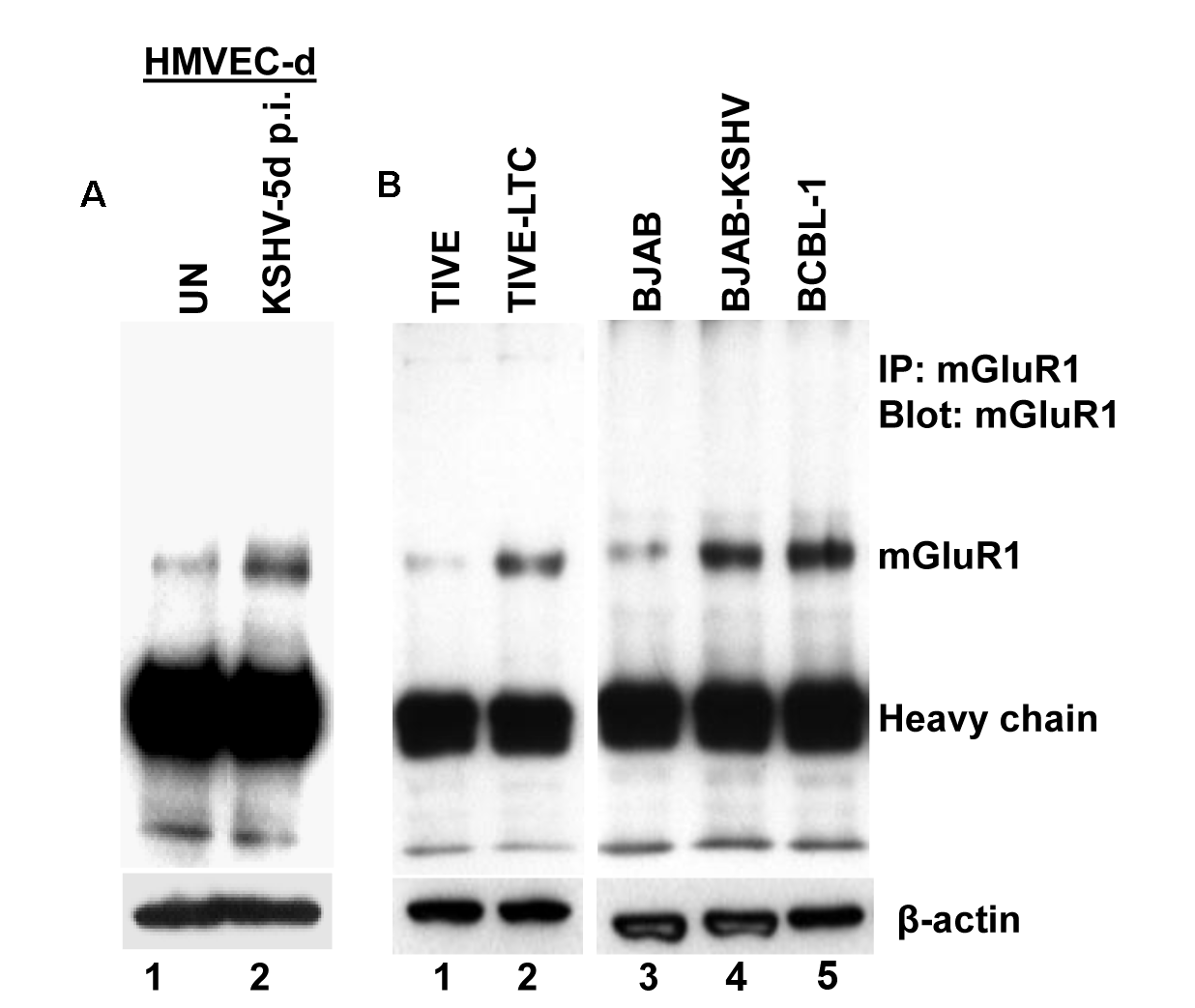

Supplement: Figure S2 — (A) HMVEC-d cells left uninfected or infected with KSHV for 5 d were immunoprecipitated using anti-mGluR1 antibodies and Western blotted with anti-mGluR1 antibody. (B) BJAB, BJAB-KSHV, BCBL-1, TIVE and TIVE-LTC cell lysates immunoprecipitated using anti-mGluR1 antibodies were Western blotted with anti-mGluR1 antibody. An equal amount of cell lysates were subjected to Western blot with β-actin used as loading control. (TIF) [file ppat.1004389.s002.tif]

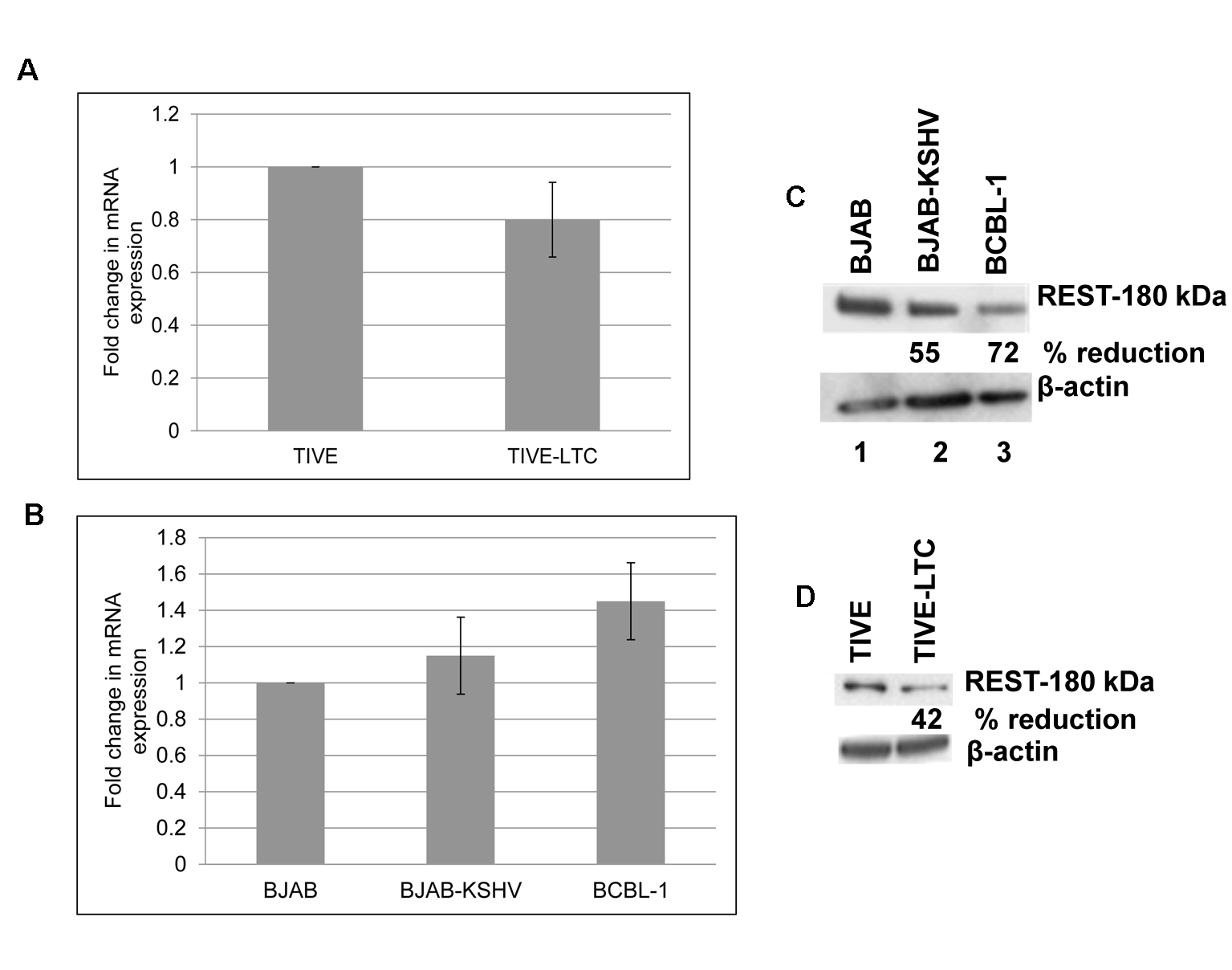

Supplement: Figure S3 — REST expression in TIVE, TIVE-LTC (A), BJAB, BJAB KSHV, and BCBL cells (B): Expression of REST mRNA assessed by quantitative RT–PCR and expressed as fold change determined using the comparative ct value based method. The fold change in gene expression is relative to the uninfected control equals 1. (C and D) Protein expression of REST in different cell lines was analyzed by immunoblotting. β-actin was used as an internal control. Percentage reduction is relative to the uninfected control. (TIF) [file ppat.1004389.s003.tif]

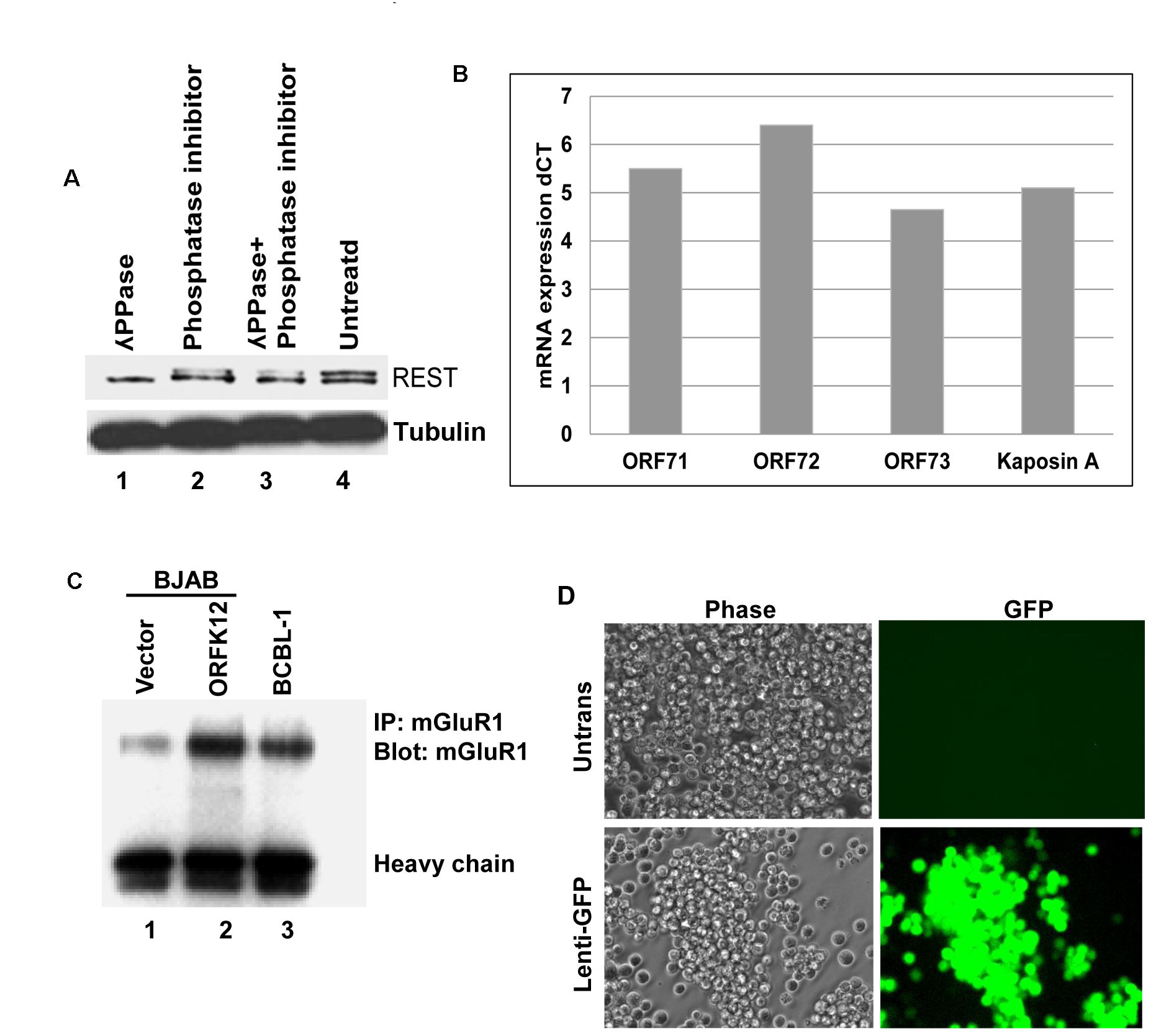

Supplement: Figure S4 — (A) Cytoplasmic extracts from TIVE-LTC cells were either left untreated or treated with λ-phosphatase or with λ-phosphatase and phosphatase inhibitors then Western Blotted with REST. Tubulin was used as loading control. Lambda phosphatase treatment was done according to the manufacturer's instructions (Santa Cruz). (B) Viral gene expression in BJAB cells: BJAB cells were transduced with the lentiviral constructs of KSHV latent ORF71, -72, -73, and –Kaposin A genes. mRNA expression of viral genes were measured by real-time PCR analysis. Results were normalized to the amount of tubulin mRNA. C) Cell extracts from the control lentivirus vector or ORFK12 transduced cells or BCBL-1 cells as a positive control were subjected to immunoprecipitation using anti-mGluR1 antibody and Western blotted with mGluR1 antibody. β-actin was used as input loading control. D) Image showing lentiviral GFP expression in BJAB cells: BJAB cells were transduced with GFP expressing lentivirus for 3 d, and the transduction efficiency was determined by observing the GFP-positive cells under an immunofluorescence microscope. (TIF) [file ppat.1004389.s004.tif]

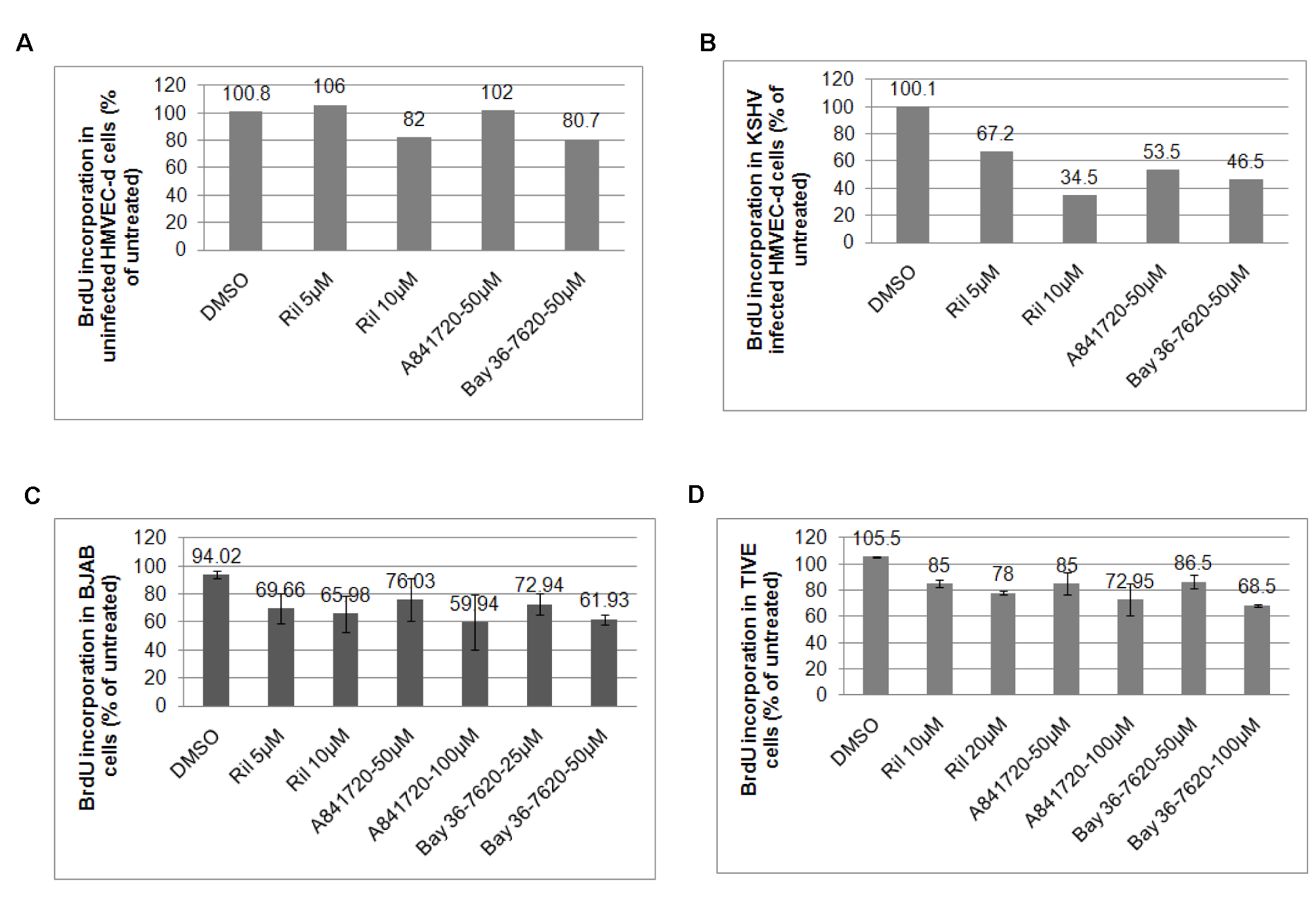

Supplement: Figure S5 — BrdU incorporation determined by ELISA in uninfected (A) and KSHV infected (B) HMVEC-d cells. HMVEC-d cells seeded in 96 well plates were left uninfected or infected with KSHV for 3 d. The cells were then cultured in the absence or presence of riluzole (5 and 10 µM), A841720 (50 µM), or Bay36-7620 (50 µM) for 48 h followed by BrdU pulse labeling for 2 h. BrdU incorporation was quantitated using a BrdU cell proliferation ELISA kit. (C and D) BrdU incorporation determined by ELISA in TIVE (C) and BJAB cells (D) treated in the absence or presence of different concentrations of riluzole, A841720, or Bay36-7620 for 48 h followed by BrdU pulse labeling for 2 h. BrdU incorporation was analyzed by a BrdU cell proliferation ELISA kit. (TIF) [file ppat.1004389.s005.tif]
